# Exosomes-Mediated Transfer of Itga2 Promotes Migration and Invasion of Prostate Cancer Cells by Inducing Epithelial-Mesenchymal Transition

**DOI:** 10.3390/cancers12082300

**Published:** 2020-08-15

**Authors:** Rofaida Gaballa, Hamdy E. A. Ali, Mohamed O. Mahmoud, Johng S. Rhim, Hamed I. Ali, Heba F. Salem, Mohammad Saleem, Mohamed A. Kandeil, Stefan Ambs, Zakaria Y. Abd Elmageed

**Affiliations:** 1 Department of Pharmaceutical Sciences, Rangel College of Pharmacy, Texas A&M Health Science Center, College Station, TX 77843, USA; Rofayda011142@pharm.bsu.edu.eg (R.G.); haali@tamu.edu (H.E.A.A.); alyismail@tamu.edu (H.I.A.); 2 Departments of Biochemistry, Faculty of Pharmacy, Beni-Suef University; Beni-Suef 62521, Egypt; mohamed.omar@pharm.bsu.edu.eg; 3 Department of Radiobiological Applications, Nuclear Research Center, Atomic Energy Authority, Cairo 13759, Egypt; 4 Department of Surgery, Uniformed Services University of the Health Sciences, Bethesda, MD 20814, USA; jrhim@verizon.net; 5 Department of Pharmaceutics, Faculty of Pharmacy, Beni-Suef University, Beni-Suef 62521, Egypt; heba_salem2004@yahoo.co.uk; 6 Department of Urology, Masonic Cancer Center, University of Minnesota, Minnesota, MN 55455, USA; msbhat@umn.edu; 7 Department of Biochemistry, Faculty of Veterinary Medicine, Beni-Suef University, Beni-Suef 62521, Egypt; mohamedkandeel561@yahoo.com; 8 Laboratory of Human Carcinogenesis, Center for Cancer Research, National Cancer Institute, NIH, Bethesda, MD 20892, USA; ambss@mail.nih.gov; 9 Department of Pharmacology, Edward Via College of Osteopathic Medicine, University of Louisiana at Monroe, Monroe, LA 71203, USA

**Keywords:** prostate cancer, CRPC, exosomes, ITGA2, metastasis, EMT, IHC

## Abstract

Although integrin alpha 2 subunit (ITGA2) mediates cancer progression and metastasis, its transfer by exosomes has not been investigated in prostate cancer (PCa). We aimed to determine the role of exosomal ITGA2 derived from castration-resistant PCa (CRPC) cells in promoting aggressive phenotypes in androgen receptor (AR)-positive cells. Exosomes were co-incubated with recipient cells and tested for different cellular assays. ITGA2 was enriched in exosomes derived from CRPC cells. Co-culture of AR-positive cells with CRPC-derived exosomes increased their proliferation, migration, and invasion by promoting epithelial-mesenchymal transition, which was reversed via ITGA2 knockdown or inhibition of exosomal uptake by methyl-β-cyclodextrin (MβCD). Ectopic expression of ITGA2 reproduced the effect of exosomal ITGA2 in PCa cells. ITGA2 transferred by exosomes exerted its effect within a shorter time compared to that triggered by its endogenous expression. The difference of ITGA2 protein expression in localized tumors and those with lymph node metastatic tissues was indistinguishable. Nevertheless, its abundance was higher in circulating exosomes collected from PCa patients when compared with normal subjects. Our findings indicate the possible role of the exosomal-ITGA2 transfer in altering the phenotype of AR-positive cells towards more aggressive phenotype. Thus, interfering with exosomal cargo transfer may inhibit the development of aggressive phenotype in PCa cells.

## 1. Introduction

Prostate cancer (PCa) is the second leading cause of cancer-related deaths among elder men [1]. Although PCa is a multifactorial disease, androgen and its receptor (AR) represent the main driving force for PCa progression. In the early stage of the disease, the cancer cells remain sensitive to androgens; therefore, androgen deprivation therapy is the most effective treatment typically offered to these category of patients [2]. Over time, however, the cancer cells become androgen insensitive, and chemotherapy is one clinical option to treat androgen-independent and metastatic castration-resistant PCa (mCRPC), a stage at which the clinical outcomes of the PCa patient are inferior [3,4]. Although circulating androgens are low, CRPC tissues are able to maintain androgen levels in such a state where AR-dependent signaling pathways still active [5]. Different mechanisms are suggested to understand this phenomenon include AR gene mutation, AR splice variant expression, AR overexpression, transcription factors overexpression, and upregulation of the androgen synthesis enzymes [6,7,8,9,10,11]. These phenotype changes warranted further studies to understand the molecular mechanisms underlying PCa progression and metastasis [12]. Undeniable, a reasonable number of men diagnosed every year with PCa will experience progression from localized to advanced metastatic state [13].

The first option for treating CRPC patients is to use anti-androgen therapies such as Enzalutamide or Abiraterone. Undesirably, up to 25% of patients develop de novo resistance against these drugs and other patients who initially responded are more likely to develop resistance within few months [14]. This suggests the urgent need for discovering new molecular mechanisms underlying these molecular changes and, therefore, develop novel treatment strategies to target this devastating disease at metastatic stage. In addition to the role of AR in the disease development, several studies reported the role of estrogen/estrogen receptor (ER) axis in PCa progression [15,16]. The possible association of ERs with epithelial-to-mesenchymal transition (EMT) could explain the reason why PCa cells become more aggressive and resistant to therapeutic targets at advanced stages [17]. Given the role of ERs in mediating EMT and PCa progression, new generations of drugs that can target EMT, ERs, and other PCa-associated nuclear factors might be an alternative option for treating mCRPC patients.

This step depends mainly on the role of intrinsic factors in neoplastic cells, such as nucleic acids and proteins that are carried by extracellular vesicles and have a significant role in cell colonization and organotropism [18]. Exosomes are bilayer membrane extracellular nanovesicles of endosomal origin that carry diverse cargo proteins, nucleic acids, and lipids. These nanovesicles can play significant roles in cells communication *via* shuttling active biomolecules into target cells. Although the role of exosomes in promoting metastasis has been established and can be targeted to reduce metastasis [19], yet the molecular mechanisms and components of exosomal cargo are still incompletely understood. For example, exosome-associated integrins play a pivotal role in pre-metastatic niche formation and organotropic metastasis [20]. This occurs by supporting metastatic dissemination through EMT and releasing autocrine and paracrine signals within the tumor microenvironment [21]. Once released into the systemic circulation, these exosomes prepare the pre-metastatic niche to receive new tumor cells, where they either remain dormant or colonize to form micro- and macrometastases [19]. While PCa cells metastasize to the bone, PCa-associated osteoblasts are playing a regulatory role in promoting steroidogenesis in CRPC cells and, therefore, maintain cell growth [22]. Thus, the idea of understanding how PCa cells become AR-independent and gain aggressive phenotypes are very significant to treat patients at the metastatic stage.

Signaling pathway mediated by integrins is considered as a mechanistic driver for the progression of PCa into metastatic disease [23], where they promote aggressive phenotypes [24]. In particular, alpha 2 integrin (ITGA2) forms a heterodimer with beta 1 subunit (α2β1) and functions as a collagen and laminin receptor [25] and is involved in the disease progression. Overexpression of ITGA2 increases cell proliferation and invasiveness of cancer cells by activation of the PD-L1/STAT3 axis [26]. In addition, ITGA2-induced chemoresistance is reversed by upregulation of miR-135b-5p, which inhibits MAPK/ERK and EMT pathways in gastric cancer cells [27]. The expression of ITGA2 is inhibited by silencing SNAIL in rhabdomyosarcoma RH30 cells and the overall metastatic behavior is reduced [28]. However, the role of exosomes-mediated transfer of integrins from CRPC to AR-dependent cells has not been investigated. Therefore, we aimed to determine the role of exosomes-mediated transfer of ITGA2 in promoting PCa migration and invasion. We found that ITGA2 was enriched in exosomes of CRPC versus AR-positive PCa cells. Co-culture of C4-2B, CWR-R1ca and RC77T/E cells with PC-3 derived exosomes promotes cell proliferation, migration, and invasion. To confirm the role of exosomal ITGA2, exosomal uptake was inhibited by MβCD and ITGA2 knockdown where the gained aggressive behavior was reversed. ITGA2 was reconstituted in two cells, which reproduced the results produced from cocultured experiments and increased cell migration and invasion.

## 2. Results

### 2.1. Characterization of Exosomes Derived From PCa Cells

**Figure 1:**
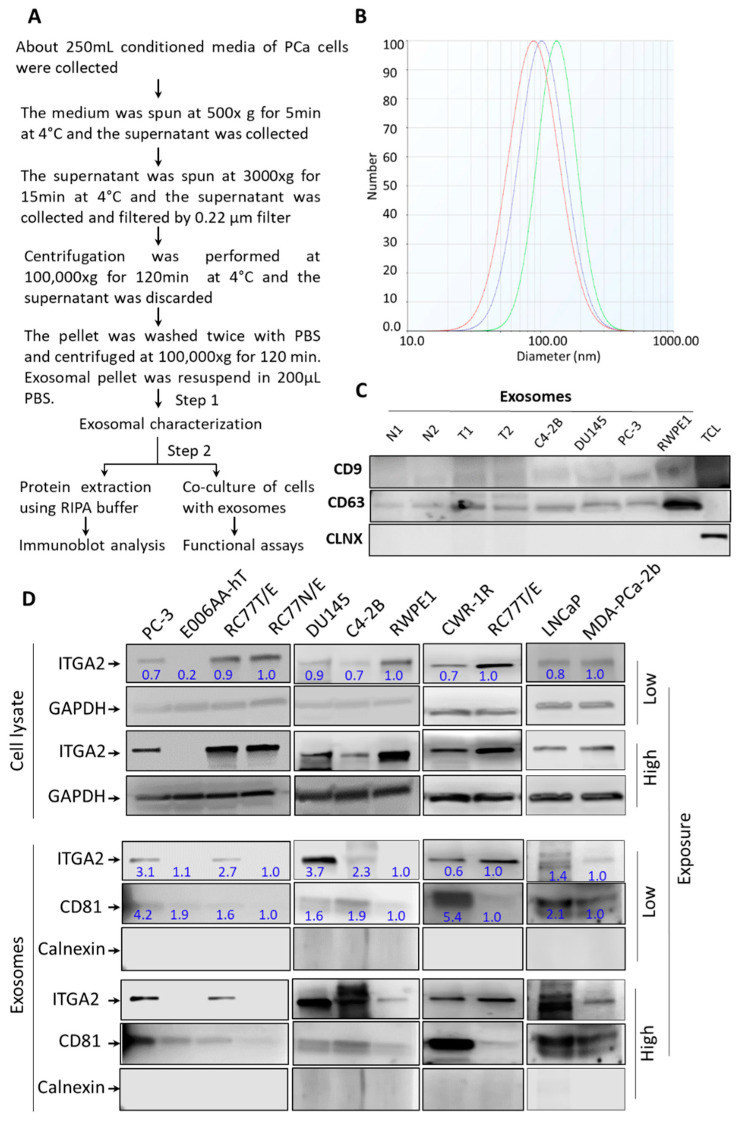
Isolation, characterization and expression of ITGA2 in exosomes derived from PCa cells. (**A**). Schematic representation of exosome isolation from PCa cells by differential ultracentrifugation. Conditioned media collected from PCa cells were used for exosomes extraction. (**B**). Zeta potential analysis was performed to determine the number, average size, and homogeneity of exosomes isolated form of PC-3 cells (*n* = 3). (**C**). Exosomes were characterized by immunoblotting (IB) analysis. About 10 µg of exosomes derived from conditioned media of cells or plasma of PCa patients (T) and normal subjects (N) in addition to total cell lysate (TCL) of LNCaP cells were loaded onto 12.5% SDS-PAGE gel. IB analysis shows the expression of calnexin as a cellular marker and CD9 and CD63 as exosomal markers. (**D**). Enrichment of exosomal content of ITGA2 was evaluated in exosomes compared to cell lysates collected from LNCaP, C4-2B, DU145, PC-3, CWR-R1ca, RC77T/E, RC77N/E, RWPE-1, MDA-PCa-2b, and E006AA-hT cells using Western blot analysis. Twenty micrograms of protein lysates were loaded, and the membranes were incubated with anti-ITGA2, anti-GAPDH, anti-CD81, and anti-calnexin (CLNX) antibodies. These experiments were repeated at least three times.

Before conducting the next experiments, the size and purity of exosomes derived from condition media of PCa cells were evaluated. Exosomes were isolated and purified by differential ultracentrifugation and then examined for their size and purity as shown in the provided flowchart (Figure 1A). A Zeta Pals Potential Analyzer (Brookhaven Instruments, Holtsville, NY, USA) was used to evaluate the size of microvesicles. The isolated exosomes from PC-3 and DU145 cells were in the range of 50 to 120 nm in diameter (Figure 1B). As depicted in Figure 1C, immunoblot analysis showed that exosomes isolated from PC-3 and DU145 cells in addition to plasma of PCa patients and their age-matched healthy individuals expressed exosomal surface marker CD9 and CD63 but not the endoplasmic reticulum marker Calnexin (CLNX). Of note, the corresponding total cell lysates expressed CLNX but not exosomal markers.

### 2.2. ITGA2 is Enriched in Exosomes Released by PCa Cells

Proteomic analysis was performed to compare exosomes isolated from PCa versus normal RWPE1 cells as previously described [29]. The results showed that ITGA2 is among top-ranked proteins expressed in exosomes derived from PCa cells. However, the role of exosomal ITGA2 in promoting PCa progression has not been investigated in this study. Therefore, this finding prompted us to examine the enrichment of ITGA2 in exosomes derived from a panel of PCa in addition to normal prostate cells. To achieve this goal, exosomes were isolated from the conditioned media of PC-3, C4-2B, DU145, CWR-R1ca, LNCaP, MDA-PCa-2b, and RC77T/E in addition to nontumorigenic prostate epithelial cells, RC77N/E and RWPE1 (Cells are described in Table S2). Recently identified renal cell carcinoma cells, E006AA and E006AA-hT, were used as control cells since they have undetectable ITGA2 expression. Results from the immunoblot analysis showed that ITGA2 was enriched in exosomes of most of metastatic PCa cells such as PC3, DU145, LNCaP, and CWR-R1 cells compared to its modest expression in normal RC77N/E and RWPE1 cells as shown in Figure 1D. Interestingly, the cellular expression of ITGA2 was distinguished in most of PCa and normal cells except LNCaP, C4-2B and MDA-PCa-2b. To study the role of exosomes-mediated transfer in altering the phenotype of PCa cells, we selected CRPC PC-3 cells as exosomal donors in most of the experiments and AR-positive LNCaP, C4-2B, and RC77T/E cells as recipient cells. Thus, LNCaP, C4-2B and E006AA-hT cells were used as a model for examining the effect of exosomes-mediated transfer and ectopic expression of ITGA2 to study the direct effect of the protein on altering traits of recipient cells. The original blots are provided in the Supplementary Figure S4A,B.

### 2.3. Exosomes-Mediated Transfer of ITGA2 and Their Uptake by PCa Cells

**Figure 2:**
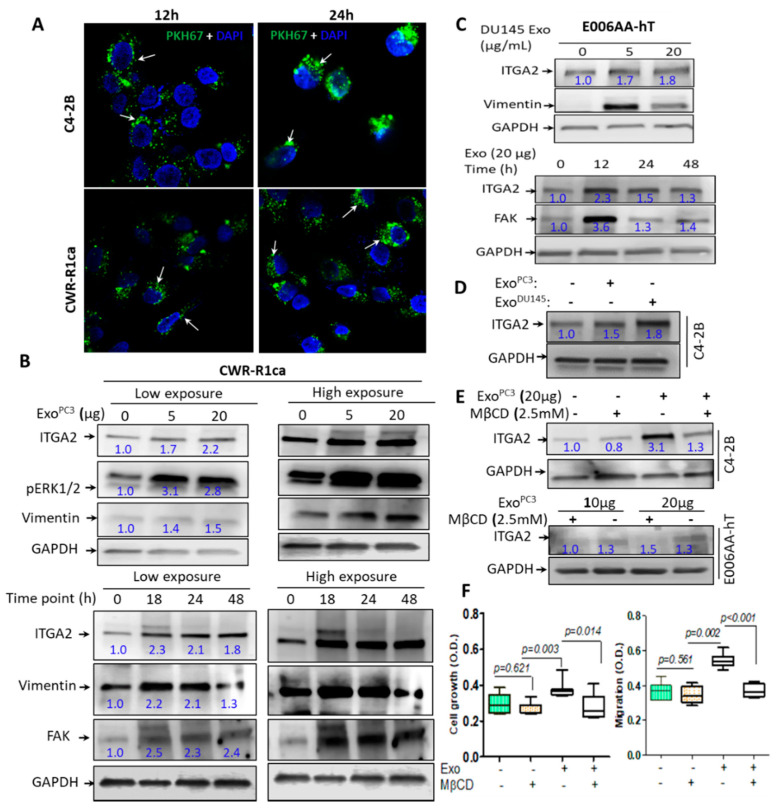
Exosomes-mediated transfer of ITGA2 in PCa cells. (**A**) Exosomal uptake of exosomes derived from PC-3 (Exo^PC3^, 10 µg/mL) labeled with PKH67 in C4-2B and CWR-R1ca cells. The green color of microbodies indicates exosomal uptake by recipient cells, and the blue color represents nuclear staining by DAPI. (**B**,**C**). Optimization of exosomes-mediated transfer in recipient cells. About 20 μg protein lysates were collected from CWR-1Rca (**B**) and E006AA-hT (**C**) cells, after their incubation with 0, 5, and 20 μg/mL Exo^PC-3^ for 48 h. Meanwhile, 20 μg protein lysate collected from CWR-1Rca and E006AA-hT cells previously incubated with 20 µg/mL Exo^PC-3^ for 18, 24, and 48 h. The membranes were probed with anti-ITGA2, anti-vimentin, anti-FAK and anti-pERK1/2 antibodies in addition to anti-GAPDH as a loading control protein. (**D**) Immunoblot (IB) analysis for C4-2B cells incubated with 20 μg/mL exo^PC-3^ for 24 h showing ITGA2 expression. (**E**) IB analysis for protein lysate collected from C4-2B and E006AA-hT cells treated without or with 2.5 mM MβCD in the presence of 20 µg/mL exo^PC-3^. (**F**) CWR-1Rca cells were treated with exo^PC−3^ with or without 2.5 mM MβCD for cell proliferation (72 h) and cell migration (24 h). The experiments were repeated at least twice. Data are statistically significant at *p <* 0.05.

To evaluate the efficiency of exosomal uptake, exosomes isolated from PC-3 cells (exo^PC-3^) were labeled with PKH67GL fluorescentyl dye. C4-2B and CWR-R1ca cells were incubated with green-labeled exosomes for 12 and 24 h and examined for the exosomal uptake by confocal microscope. The results showed a remarkable uptake of labeled exosomes as shown by the green-fluorescent dots that appeared in the cytoplasm of the recipient cells (Figure 2A). To address whether exosome-associated ITGA2 derived from PC-3 cells will be transferred to other recipient cells with low ITGA2 expression, CWR-R1ca and control E006AA-hT cells were incubated with two different concentrations of 5 and 20 µg/mL of exo^PC-3^ and at different time points (from 18–48 h). The immunoblot results demonstrated a significant increase in ITGA2 expression after incubation of cells with 5 and 20 µg/mL exo^PC-3^ and as early as 18 h and up to 48 h incubation period compared to control cells (Figure 2B,C). As a result of exosomal transfer of ITGA2, the expression of Vimentin as EMT marker and FAK was also increased. Meanwhile, the activity of ERK1/2 was increased regardless the concentration of exosomes.

These optimized conditions (5 µg/mL exosomes and 24 h incubation time) were established in C4-2B cells (Figure 2D). To exclude other possible sources of ITGA2 other than exosomes, we incubated the recipient CWR-R1ca and E006AA-hT cells with 20 µg/mL exo^PC-3^ for 18 h in presence or absence of MβCD (2.5 mM) as an exosomal-uptake inhibitor. The results showed that CWR-R1ca and E006AA-hT cells treated with MβCD followed by incubation with 20 µg/mL of exo^PC-3^ had low expression of ITGA2 compared to control cells, which either received the inhibitor alone or with no treatment (Figure 2E). The inhibitory effect of MβCD was also reflected on functional activities of CWR-R1ca cells where the inhibitor suppressed cell growth and migration compared to control cells (Figure 2F). The original blots are provided in the Supplementary Figure S5A–C.

### 2.4. Knockdown of ITGA2 in CRPC Cells Decreases the Regulatory Effect of Exosomes on Recepient Cells

**Figure 3:**
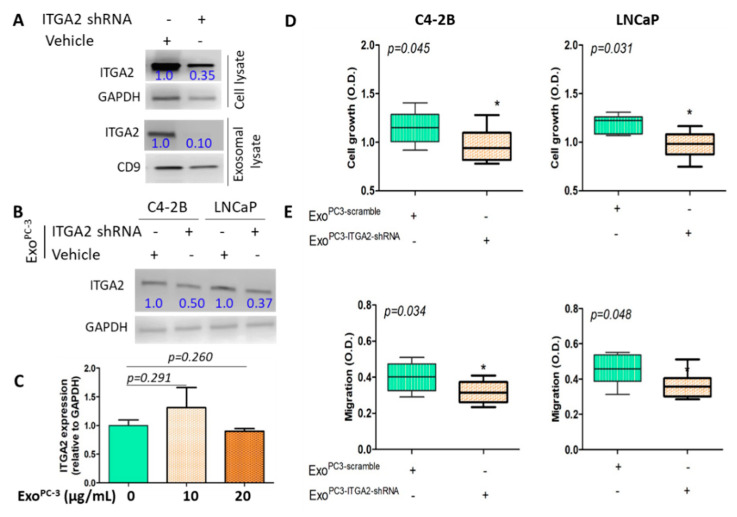
Knockdown of ITGA2 in PC-3 cells suppresses the effect of exosomes on recipient C4-2B and LNCaP cells. (**A**). PC-3 cells were transduced with shRNA specific to ITGA2 in addition to scrambled control. Immunoblot (IB) analysis was performed to examine the expression of ITGA2 in total cell and exosomes lysates. (**B**). C4-2B and LNCaP cells were incubated with 20 µg/mL exosomes derived from ITGA2 knockdown PC-3 cells. (**C**) C4-2B cells were incubated with 10 and 20 µg/mL exosomes derived from ITGA2 knockdown PC-3 cells for 24 h. RNA was extracted and real-time PCR was performed to determine the expression of *ITGA2* relative to *gapdh*. (**D**,**E**) C4-2B and LNCaP cells were incubated with exosomes isolated from the integrin knockdown PC-3 cells and cell proliferation and migration assays were assessed. Data considered significant at *p <* 0.05. The experiment was repeated twice.

To examine the possibility that the cellular effect in recipient cells was triggered via transfer of CRPC exosome-associated ITGA2 and was not originated from endogenous expression of ITGA2, we transduced PC-3 cells with lentivirus carrying shRNA specific to ITGA2. After 72 h of transduction, immunoblot analysis show a significant decrease in the expression of ITGA2 on cellular and exosomal levels (Figure 3A). Further, incubation of C4-2B and LNCaP cells with exo^PC−3^-bearing ITGA2-shRNA suppressed the expression of ITGA2 compared to cells received vehicle only (Figure 3B). On functional level, cell growth and migration decreased in the C4-2B and LNCaP cells in comparison to control cells as shown in Figure 3D,E. As a second step, C4-2B cells were treated with exo^PC−3^ at two different concentration (10 and 20 µg protein/mL) for 24 h. The original blots are provided in the Supplementary Figure S5D.

The results from qPCR analysis show that there was no significant difference between control and exo^PC-3^-treated cells (Figure 3C). This may rule out the endogenous expression of ITGA2 via cell machinery, which was not detected by exosomal-associated ITGA2 uptake during this time window.

### 2.5. Exosomes-Mediated Transfer of ITGA2 Increased Cell Proliferation, Migration, and Invasion, and Reduced Cell Adhesion

**Figure 4:**
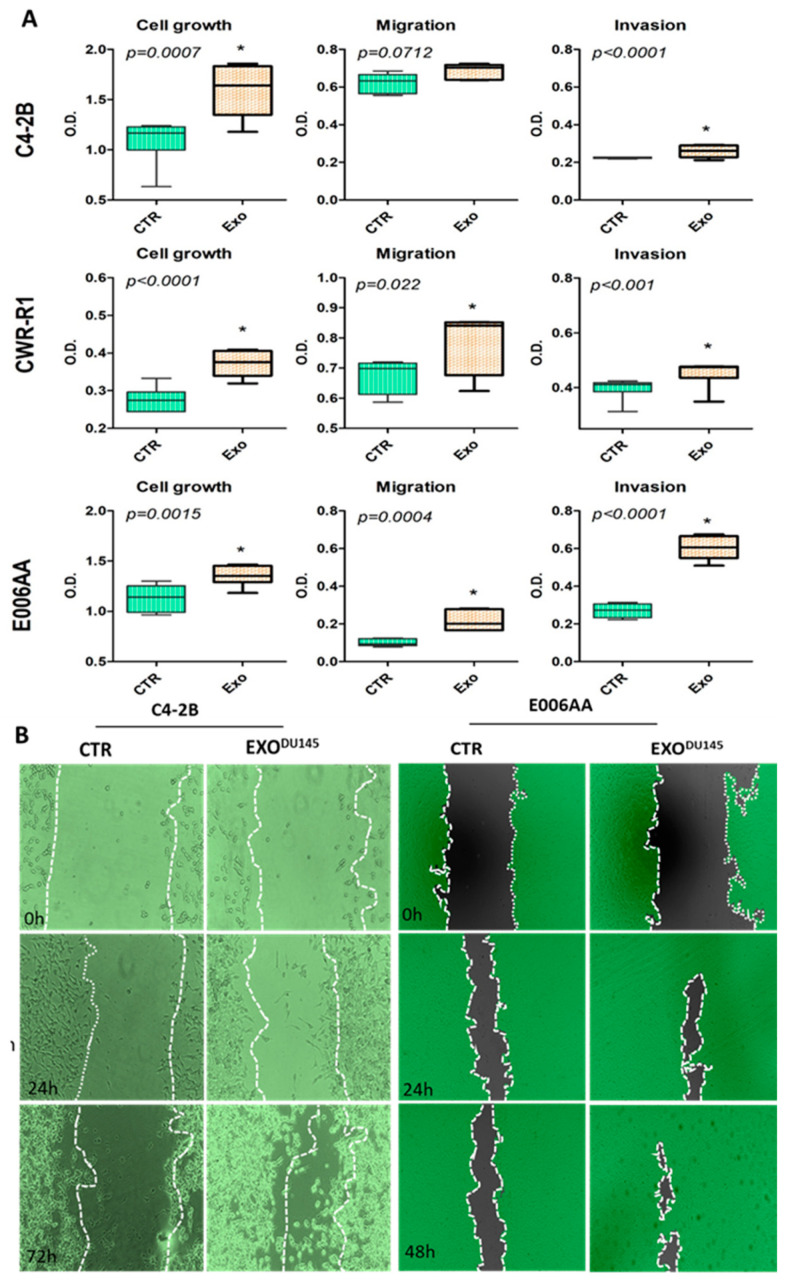
Functional significance of exosomes-mediated transfer of ITGA2 into PCa cells. (**A**) C4-2B, CWR-1Rca and E006AA cells were incubated with 10 µg/mL exosomes derived from PC-3 or DU145- for 3 days then cell proliferation assay was performed. Representative transwell migration and invasion assays are presented for C4-2B, CWR-1Rca and E006AA cells treated with or without 20 µg/mL exo^PC-3^ for 24 h. (**B**) Wound-healing assay was performed on C4-2B and E006AA cells incubated with or without exosomes derived from DU145 cells up to 72 h in 6-well plates. Each treatment was conducted in triplicate and at least 6 fields were captured under inverted bright microscope. * depicts significance at *p* < 0.05. Each experiment was performed in triplicate and independently repeated thrice.

To evaluate the functional significance of exosomal ITGA2 in mediating PCa progression and metastasis, C4-2B, CWR-R1ca and E006AA cells were incubated with exo^PC-3^ for 72 h (cell growth) and for 24 h (migration and invasion assays). The results revealed a significant increase in cell proliferation (*p* ≤ 0.0015) in the three cells versus their counterparts (Figure 4A). Meanwhile, we reproduced the same experiment in RC77T/E and E006AA-hT cells and their growth rate was also increased (*p* < 0.001) as shown in Supplementary Figure S1. In addition, C4-2B, CWR-R1ca and E006AA cells had higher migration (*p* < 0.05) and invasion (*p* < 0.001) except for migration of C4-2B (*p* = 0.071) when these cells were incubated with 10–20 μg/mL exo^DU145^ as compared to control cells (Figure 4A and Supplementary Figure S3A). Similarly, wound-healing assay resulted in an increase of migration of C4-2B and E006AA cells in presence of exo^DU145^ compared to control cells (Figure 4B). Consistent with our results, cell adhesion assay showed that the C4-2B and E006AA cells co-cultured with exo^DU145^ had low attachment capabilities (*p* < 0.05) regarding control cells (Supplementary Figure S1).

### 2.6. Ectopic Expression of ITGA2 in C4-2B Cells Reproduces the Effect of Exosomes-Mediated Transfer of ITGA2

**Figure 5:**
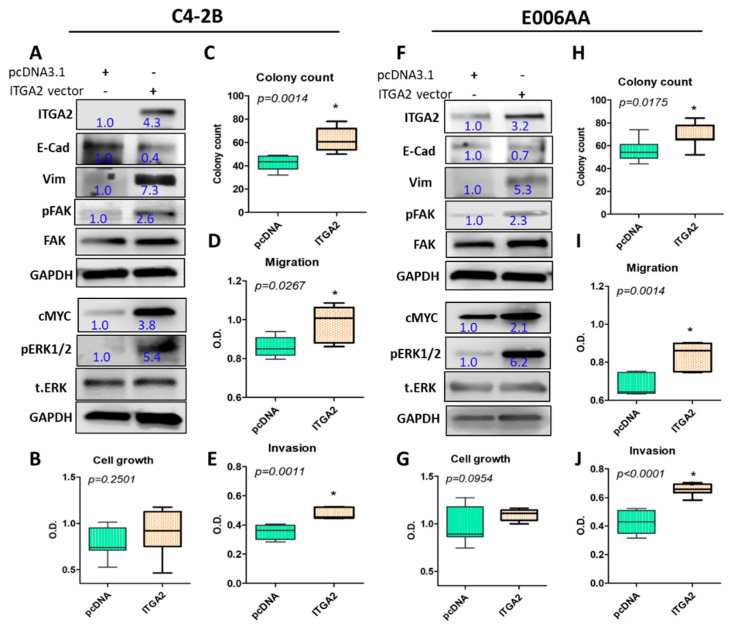
Ectopic expression of ITGA2 promotes aggressive phenotypes in PCa cells. C4-2B and E006AA cells were transfected with 2.5 µg plasmid carrying ITGA2 or pcDNA3.1 as a control plasmid for 96 h. The expression of ITGA2, E-cadherin (E-Cad), vimentin (Vim), and c-Myc, and the activity of FAK and ERK1/2 were evaluated in these cells (**A**,**F**). Cell proliferation (**B**,**G**) and clonogenic (**C**,**H**) assays in the ITGA2-transfected cells were performed. Transwell migration (**D**, **I**) and invasion (**E**,**J**) assays were also conducted over a period of 18 to 48 h. * depicts significance at *p* < 0.05. Each experiment was performed in triplicate and independently repeated three times.

To confirm the favorable effect resulted from exosomes-mediated transfer of ITGA2, C4-2B and E006AA cells were transfected with ITGA2-mCherry vector in addition to pcDNA3.1 as a vehicle control. Ectopic expression of ITGA2 in C4-2B and E006AA was validated by Real-Time PCR (Supplementary Figure S3B) and Western blot analysis (Figure 5A,F). Noteworthy, overexpression of ITGA2 augmented the stemness of cells by increasing mesenchymal marker Vimentin in both C4-2B and E006AA and decreased the epithelial marker E-cadherin. Likewise, cMYC expression, and phosphorylation of FAK and ERK1/2 were increased in C4-2B and E006AA cells (Figure 5A,F). As shown in Figure 5B,G, the ectopic expression of the integrin in the two cells exhibited no significant effect on cell proliferation. However, it increased the number of colonies (*p* < 0.05) in the ITGA2-bearing cells (Figure 5C,H and Supplementary Figure S3C). Moreover, ITGA2-expressing cells induced higher cell migration and invasion (*p* < 0.05) as shown in Figure 5D,E,I,J and increased migratory cells (Figure S2) compared to control cells. The original blots are provided in the Supplementary Figure S6.

### 2.7. Expression of ITGA2 in Human PCa Tissues

**Figure 6:**
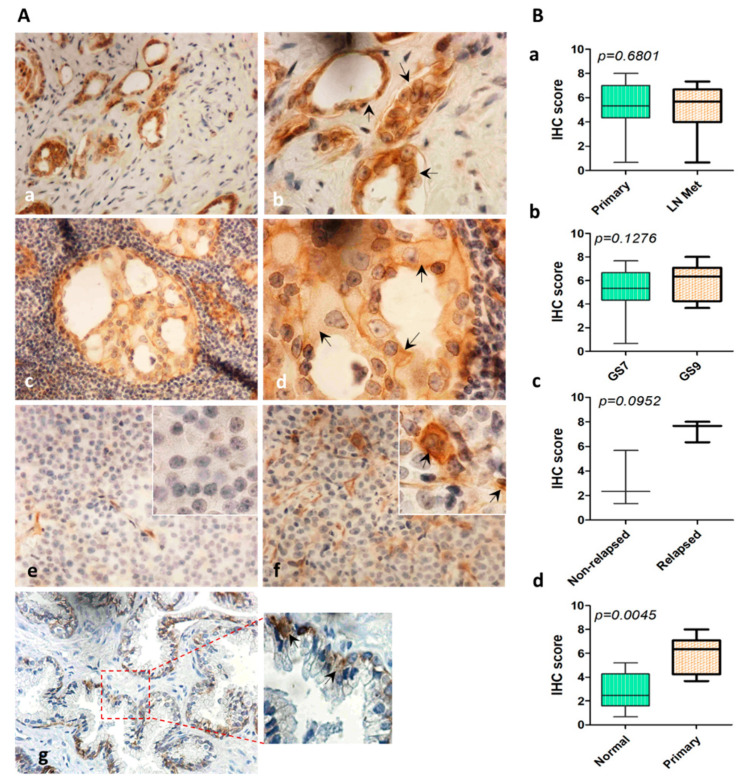
Expression of ITGA2 in PCa tumors with lymph node metastasis. Tissue cores were stained with anti-ITGA2 antibody. (**A**) Immunostaining of PCa tissue cores of primary (**a**, **b**) and lymph node metastasis tumors (**c**,**d**). Expression of ITGA2 was evaluated in non-relapsed (**e**), relapsed (**f**), and normal (**g**) tissue specimens. (**B**) Immunohistochemical score of ITGA2 in primary versus tumors with lymph node metastasis (**a**). High Gleason score (GS9) versus low Gleason score (GS7) (**b**). Relapsed versus non-relapsed tumors (**c**), and normal versus primary tumors (**d**). Magnification was 400× (**a**, **c**, **e**–**g**) and 1000× (**b**,**d** inserts).

The expression of ITGA2 was evaluated in 48 PCa tissue cores; 24 primary tumors in addition to their matched metastatic lymph node tissues. Another five healthy prostate tissues were stained. As shown in Figure 6, ITGA2 was mostly expressed in the cytoplasm and the expression was increased in primary tumor compared to normal tissues (*p* < 0.0045). However, there was no significant difference in the integrin expression between the primary and lymph node metastatic tumors (*p* = 0.7858). When Gleason score (GS) was considered, lymph node metastatic tissues of GS = 9 had a slight increase in ITGA2 expression (*p* = 0.1275) compared to other tissues collected from PCa patients at GS = 7. Of note, high expression of the integrin was observed in relapsed versus non-relapsed tumors (*p* = 0.0952).

### 2.8. Expression of ITGA2 in Exosomes Isolated From the Plasma of PCa Patients

**Figure 7:**
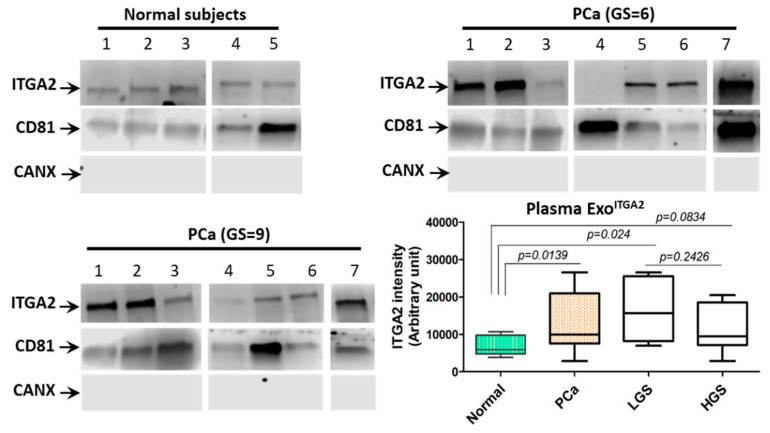
Expression of ITGA2 in exosomes isolated from plasma collected from PCa patients. (**A**) Exosomes were isolated from plasma of 14 PCa patients in addition to five healthy subjects. Exosomal protein lysates were prepared and immunoblot analysis was performed using anti-ITGA2, anti-CD81, and anti-Calnexin antibodies. The original immunoblots are provided in the Supplementary Materials. (**B**) Quantification of ITGA2 enriched in exosomes isolated from plasma of PCa patients and healthy subjects as a control group.

To validate our findings in PCa blood samples, we isolated exosomes from blood plasma of 14 PCa patients in addition to six healthy subjects as a pilot study. As depicted in Figure 7, exosomal ITGA2 was highly enriched (*p* = 0.0139) in the plasma procured from PCa patients compared to age-matched healthy subjects. Interestingly, exosomes collected from the plasma of patients with low GS had a slight increase in ITGA2 enrichment when compared to exosomes collected from patients with high GS (*p* = 0.2426). However, exosomes collected from the plasma of patients with low GS had more enriched integrin than normal subjects (*p* = 0.024). To confirm the purity of exosomes, CD81 was used as an exosomal marker and calnexin a cellular marker. The original blots are provided in the Supplementary Figure S7.

## 3. Discussion

The development of castration-resistant PCa (CRPC) is a current major concern in PCa management where most of patients develop metastasis. The importance of cellular crosstalk between PCa cells and to their tumor microenvironment (TME) to promote progression and metastasis has been documented in cancer research. Although a number of studies suggested the role of soluble factors in modulating TME, the potential role of exosomes in altering TME to induce tumor aggressiveness is gaining momentum [30,31]. Understanding the underlying mechanisms and cargo contents of exosomes-mediated transfer to and from PCa cells and TME at advanced stages of the disease will constitute a strong foundation on which novel and more specific therapeutic targets may be evolved.

Our results show that ITGA2 was enriched in exosomes of most of CRPC versus AR-positive PCa cells. Interestingly, co-culture of PC-3- or DU145-derived exosomes with either C4-2B, LNCaP, RC77T/E and E006AA cells promotes cell proliferation, migration and invasion capacities. Although a recent study reported that E006AA-hT cells are not African American PCa cells and, instead, they have 92% similarity to European ancestry and are 86% similar to renal cell carcinoma [32], these cells were used as a control recipient cells because they have low ITGA2 expression on cellular and exosomal levels. COS-1 and CV1 cells are kidney cells used as controls in a number of PCa studies [33,34]. Meanwhile, our study utilized other authenticated PCa cells as recipient cells for exosomes uptake such as C4-2B, LNCaP, RCC7T/E, and CWR-R1ca. When C4-2B, LNCaP and E006AA cells were incubated with exosomal uptake inhibitor MβCD or exosomes partially depleted from ITGA2 using shRNA knockdown approach, the acquired aggressive behavior induced by metastatic PCa-associated exosomal ITGA2 was suppressed. To confirm the role of ITGA2-enriched in exosomes, ectopic expression of ITGA2 was established in two cells; C4-2B and E006AA where they have low expression of ITGA2, which resulted in a significant increase in migration and invasion. In addition, induction of ITGA2 increased the expression of EMT marker vimentin and ITGA2-associated downstream proteins while decreased the expression of E-cadherin. The aggressive behavior exhibited by incubating AR-positive cells with exosomes enriched with ITGA2 occurred in a very short time (12–24 h) compared to the prolonged time needed to exert the effect originated from ectopic expression of ITGA2 (at least 48 or 72 h). This process is relevant to the period of time through which exosomes can be internalized by recipient cells. Therefore, our findings anticipate that exosomal-mediate transfer of ITGA2 from CRPC cells promoted, in part, the aggressive behavior of AR-positive PCa cells through the EMT pathway. Comparing primary tumors with their matched metastatic lymph node tumors showed no significant difference in ITGA2 expression. Notably, the expression of ITGA2 at a high Gleason score was relatively high in lymph node metastasis and in relapsed versus non-relapsed tumors. However, the number of tumor specimens was limited to segregate any possible variations. Generally, exosomal ITGA2 was highly enriched in the sera procured from PCa patients who had low GS compared to high GS and normal subjects.

A growing body of evidence suggests that integrins are important components in modulating tumor progression and metastasis [35]. It was reported that αvβ6 integrin is-associated with CRPC development by the activation of the JNK1/AR axis [36]. ITGA2 is overexpressed in a number of malignant cells and its inhibition induces apoptosis and decreases cell migration [37]. Its overexpression is also distinguished in highly metastatic PCa cell lines [38]. ITGA2 regulates cells adhesion and invasion via activation of FAK, src, paxillin, Rac, and JNK pathways, and simultaneously increases the activity of matrix metalloproteinase-2 [39]. We report that ITGA2 was mostly identified in exosomes isolated from PC-3, DU145, CWR-R1ca, and RC77T/E cells compared to normal prostate cells. In agreement with our data, a number of studies showed that integrins are enriched and transferred by exosomes from PCa cells to other recipient cells. For example, αvβ3 integrin was enriched in exosomes isolated from plasma collected from PCa patients [40]. Another study shows that exosomes transfer αvβ6 from PCa cells to monocytes to induce their M2 polarization [41]. Additionally, αvβ3 integrin was shuttled from PC-3 and CWR22PC to BPH-1 and C4-2B cells and promotes cell migration [42]. In other fluids, ITGA3 was more enriched in exosomes collected from urine of metastatic PCa patients compared to non-metastatic patients and benign prostatic hyperplasia [43]. The most critical step in the transfer of exosomal cargo is the internalization of exosomes into recipient cells and blocking this critical step via disruption of exosomes lipid rafts can suppress the exosomal uptake [44,45]. Our findings show that exosomes derived from PC-3 and DU145 cells were internalized into C4-2B, LNCaP and E006AA cells and blocking exosomes uptake by MβCD inhibited cell growth and migration. Knockdown of ITAG2 in PC-3 cells was reflected in the exosomal cargo and has shown to decrease cell proliferation and migration. Co-culture of C4-2B and E006AA cells with exosomes-enriched with ITGA2 increased the rate of cell growth, migration, and invasion, and decreased cell adhesion. The insignificant increase in growth rate of C4-2B compared to E006AA cells could be explained by the different duplication time of the two cells and the rate of exosomal uptake. It was reported that co-culture of PCa exosomes with stromal cells had no effect on cell growth but rather stimulated their migration [46]. The transfer of hyaluronidase-1 from PCa-associated exosomes into prostate stromal fibroblasts increased their migration [46]. The increase in cell migration was associated with a high propensity of fibroblasts to adhere to Type IV collagen along with FAK activation. Dai and coworkers demonstrated that PCa-derived exosomes enriched with pyruvate kinase M2 prompts bone marrow stromal cells to generate premetastatic niches in the bone tissue [47].

Strikingly, establishment of ectopic expression of ITGA2 in C4-2B and E006AA cells confirmed the aggressive phenotype acquired by AR-positive cells as a result of exosome-mediated transfer of ITGA2 and corroborated by exosome co-culture experiments. This finding adds another line of evidence that exosome-associated ITGA2 is a contributing factor to PCa progression and metastasis. This notion was corroborated by a decrease in E-cadherin, and an increase in vimentin, FAK activity and c-Myc expressions. Meanwhile, the phosphorylation of ERK1/2 was augmented compared to the control cells. A number of studies reported that integrins promote cancer metastasis through epithelial-mesenchymal transition (EMT) [48,49], which are in line with our findings. The IHC data revealed no difference in the expression of ITGA2 in primary and tumor tissues with lymph node metastasis. However, we observed a trend of increase at Gleason score 9 and in tissues collected from relapsed PCa patients. The expression of ITGA2 in PCa primary versus metastatic tissues are still controversial. It has shown that primary PCa tissues had higher ITGA2 expression compared to lymph node metastasis [50]. However, Bonkhoff and coworkers [51] reported that PCa lymph node metastasis had higher ITGA2 expression compared to control tissues. In addition, the expression of ITGA2 in tissues collected from breast cancer patients was lower in lymph node-positive compared to lymph node-negative metastases [52]. Furthermore, in gastric cancer, a high expression of ITGA2 was distinguished in metastatic compared to primary gastric cancer tissues and the integrin was correlated with poor prognosis [53]. This variation in the expression pattern of ITGA2 across different studies may originate from using different types of antibodies and scoring system in addition to tumor heterogeneity of tissue specimens [54,55]. Moreover, the expression of integrins depends on the cell type and context [56]. The results also showed that circulating exosomes derived from PCa patients are enriched with ITGA2. This was evidenced by another study in which αvβ3 integrin was detected in exosomes collected from blood of PCa patients [40]. In a preclinical PCa model, it has been shown that the αvβ6/JNK1/AR axis is an important contributing factor in promoting CRPC progression [36].

## 4. Materials and Methods

### 4.1. Cell Culture

The human PCa cell lines C4-2B, DU145, PC-3, LNCaP, MDA-PCa-2b, and normal prostate RWPE-1 cells were purchased from American Type Culture Collection (ATCC, Manassas, VA, USA). CWR-R1ca cells were purchased from Millipore Sigma (Burlington, MA, USA). E006AA cells are also available from Millipore Sigma but recent report revealed that E006AA-hT were 86% closer to renal cell carcinoma [32]. These cells were cultured as we previously described [57]. Briefly, cells were maintained in either DMEM or RPMI 1640 medium supplemented with 10% fetal bovine serum and 1% penicillin/streptomycin (Life Technologies Corp., Grand Island, NY, USA). However, MDA-PCa-2b cells were kept in HPC1 medium (Athena Environmental Sciences Inc., Baltimore, MD, USA) that supplemented with 20% FBS and 50 µg/mL G418. RC77T/E PCa cells and RC77N/E normal prostate cells were provided by Dr. J.S. Rhim (Uniformed Services University) and maintained as described [57]. All cells were maintained at 37 °C and 5% CO_2_ and tested for mycoplasma every six months.

### 4.2. Isolation and Purification of Exosomes From PCa Patients and Cells

All research activities were performed in accordance with the guidelines of a protocol approved by the Institutional Review Board (IRB) from the National Cancer Institute, NIH, Bethesda, MD and Texas A&M Health Science Center (IRB#2017-0190M), College Station, TX. Informed consent was obtained from all PCa patients involved in this study prior of conducting the study. About 300 µL of plasma collected from twenty samples (14 PCa and six age-matched healthy individuals) was mixed with 3 µL of 500 U/mL Thrombin and incubated for 5 min at room temperature (Qiagen, Germantown, MD, USA). After centrifugation at 10,000× *g* for 5 min, the supernatant was collected and subjected to exosomes isolation steps. The clinical characteristics of these samples are provided in Supplementary Table S1. The ExoQuick^®^ UTRA EV Isolation kit (System Biosciences, Palo Alto, CA, USA) was utilized to purify plasma exosomes according to the manufacturer’s protocol. Briefly, about 250 µL plasma was centrifuged at 3000× *g* for 15 min. The supernatant was collected to new tubes and centrifuged again at 12,000× *g* for 15 min. The supernatant was collected and 67 µL of ExoQuik reagent was added and incubated overnight at 4 °C. The ExoQuik/biofluid mixture was centrifuged at 3000× *g* for 10 min. The supernatant was carefully aspirated and exosomal pellets were resuspended in 200 µL buffer B and purified using purification columns.

Exosomes were isolated from conditioned media of cell lines by differential ultracentrifugation 36–48 h after replenishing the medium with new medium contains exosomes-depleted FBS. Briefly, conditioned medium was collected and centrifuged at 500× *g* for 5 min and the supernatant was then collected and re-centrifuged at 3000× *g* for 15 min. The debris-free supernatant was filtered and spun down in ultracentrifuge tube at 100,000× *g* in a 45Ti rotor (Beckman Coulter, Indianapolis, IN, USA). The pellet was recovered after 2 h of ultracentrifugation and washed in PBS followed by a second spin at 100,000× *g* for 2 h. Finally, exosomal pellet was resuspended in 200 µL PBS and either used immediately or kept at −80 °C for further analyses. 

### 4.3. PKH67-Labeled Exosomes Uptake

Exosomes derived from PC-3 cells were labeled with PKH67 dye (Sigma-Aldrich, Saint Louis, MO, USA) according to the manufacturer’s protocol with minor modifications. Briefly, PC-3-associated exosomes were re-suspended in the dye mix and incubated at room temperature for 3 min. The reaction was stopped by adding 500 μL of 1% BSA in 1X PBS. Labeled exosomes were centrifuged at 100,000× *g* for 1 h. C4-2B or CWR-R cells were serum-starved for 8 h, and then incubated with or without the labeled exosomes (10 mg/mL exo^PC-3^). After 24 h, cells were fixed in 4% paraformaldehyde then stained with DAPI and photographed using an Eclipse 80i microscope (Nikon Instruments, Melville, NY, USA).

### 4.4. Exosome Uptake Inhibition and Knockdown of ITGA2

Recipient cells were treated with or without 2.5 mM methyl-β-cyclodextrin (MβCD) for 30 min before incubation of cells with 20 µg/mL exo^PC-3^. The concentration used was as previously described [45,58]. Cells were treated with or without 2.5 mM MβCD as controls. After 12 h of incubation of recipient cells with exosomes, protein lysates were collected. In another experiment, PC-3 cells transduced with either lentiviral particles carrying shRNA specific to *ITGA2* or scrambled for 72 h following the manufacturer’s instructions (Santa Cruz Biotechnology, Dallas, TX, USA) and cell and exosomal lysates were collected. ITGA2 expression was assessed by Western blot analysis. In addition, C4-2b and LNCaP cells were incubated with exosomes derived from ITGA2 knockdown PC-3 cells for 72 and 24–48 h for performing cell proliferation and migration assays, respectively.

### 4.5. Transfection of PCa Cells

Cells were seeded in 6-well plates a day before transfection. Cells were transfected with 2.5 µg DNA vectors carrying DYKDDDDK-ITGA2 (GeneCopoeia, Rockville, MD, USA), mCherry ITGA2 (Addgene, Cambridge, MA, USA) or pcDNA3.1 as an empty control plasmid using Lipofectamine 3000 (Invitrogen, Carlsbad, CA, USA) following the manufacturer’s protocol. The culture medium was removed 6 h after transfection and replenished with complete medium for another 48 h. Trypsinized cells were counted and applied for different functional assays in addition RNA and protein lysate collections for qPCR and Western blot analyses.

### 4.6. Real-Time PCR Analysis

RNA was extracted from either C4-2B cells incubated with exo^PC-3^ or exosomes isolated from *ITGA2* knockdown PC-3 cells in addition to the transfected cells with *ITGA2* using Trizol reagent according to the manufacturer’s instructions (Invitrogen Corp., Carlsbad, CA, USA). cDNA was prepared using iScript ^TM^ cDNA synthesis kit (Bio-Rad Laboratories, Hercules, CA, USA) and qPCR was performed using SYBR Green master mix (Bio-Rad Laboratories, Hercules, CA, USA) on a Bio-Rad CFX96 detection system. Conventional PCR was also performed to optimize and guarantee the specificity of the *ITGA2* primer. The fold change of gene expression in the two set of experiments was calculated compared to β-actin and 5SrRNA by comparing Ct method as described [57].

### 4.7. Western Blot Analysis

PCa cells and exosomal lysates were prepared using RIPA buffer (Santa Cruz Biotechnology, Inc., Dallas, TX, USA) and total proteins were quantified using bicinchoninic acid (BCA) assay reagents (Thermo-Scientific, Rockford, IL, USA). About 10–20 μg protein lysates were fractionated on 4–20% SDS-PAGE. Proteins were then transferred into nitrocellulose membranes (Bio-Rad, Hercules, CA, USA), blocked in 5% BSA for 1h at room temperature. Membranes were incubated overnight at 4 °C with anti-integrin α2 (ITGA2), anti-CD9, anti-CD63, anti-E-cadherin, and anti-calnexin (GeneTex, Irvine, CA, USA), anti-c-Myc, anti-phosphorylated and total FAK, anti-GAPDH (Santa Cruz Biotechnology, Dallas, TX, USA), anti-Vimentin (Millipore), anti-pERK1/2 (Cell Signaling, Danvers, MA, USA) antibodies. Membranes were incubated with appropriate secondary antibody for 1 h at room temperature. The specific protein bands were developed using the Clarity ^TM^ Western ECL Substrate (Bio-Rad Laboratories, Hercules, CA, USA) according to the manufacturer’s instructions. The developed signals were visualized by Odyssey ^®^ Fc Imager and C-Digit Blot Scanner (LI-COR, Lincoln, NE, USA) and the densitometric analysis was performed by the Image studio Lite (LI-COR, Lincoln, NE, USA).

### 4.8. Cell Proliferation Assay

C4-2B, LNCaP, CWR-R1ca and E006AA-ht cells incubated with 10 µg/mL exosomes or ITGA2-transfected cells were seeded in 96-well plate (2000 cells/well). Cells were incubated for 72 h and 10 µL of WST-8 reagent was added and cells were incubated for 2 h at 37 °C following the manufacturer’s protocol (Cell Counting Kit-8, Dojindo Molecular Technologies, Inc., Rockville, MD, USA). The developed signal was measured at 450 nm by accuSkan FC plate reader (Fisher Scientific, Hampton, NH, USA).

### 4.9. Colony-Formation Assay

Cells were seeded in six-well plate (100 cells/well) in 2 mL of complete medium containing 10% FBS and kept for 10–14 days. Cells were washed with PBS and fixed with 4% paraformaldehyde for 10 min. The growing colonies were stained with 1% crystal violet (Hardy Diagnostics, Santa Maria, CA, USA) for 30 min and the stained colonies were counted and photographed.

### 4.10. Migration and Invasion Assays

Cell migration and invasion assays were performed in 24-well plate using transwell cell culture chambers 8-μm sized pores coated with BD Matrigel^TM^ (BD Biosciences, San Jose, CA, USA) in invasion or uncoated in migration assay. Briefly, about 0.5–1.0 × 10^5^ cells were suspended in serum-free medium provided with 0.1% BSA and seeded in the upper transwell chamber. DMEM containing 10% FBS were added underneath the insert and incubated at 37 °C in 5% CO_2_ humidified chamber for 16–48 h according to the cell type. Non-migrated cells in the upper chamber were scrubbed with a cotton-tip swab, and the migrating cells were fixed in 4% paraformaldehyde and stained with 0.5% crystal violet. The insert washed twice in water then dried out. The migrated cells were counted from at least five random fields (magnification 100×) per each well then photographed under light microscope. In addition, the dye of the migrated cells was dissolved in 30% acetic acid and measured at 540 nm.

### 4.11. Immunohistochemical (IHC) Studies

IHC staining with anti-ITGA2 antibodies (Invitrogen, Carlsbad, CA, USA) was performed as previously described [59]. Briefly, normal prostate tissue slides in addition to tissue microarray slide encompassing 48 cores of PCa primary and its matched lymph node metastasis (TriStar Tech. Group, Washington, DC, USA) were de-waxed in xylene, rehydrated in descending series of alcohol and heated in 0.01M Citrate buffer solution (pH 6) for 20 min. To block the endogenous activity of peroxidase, the slides were incubated 10 min in 3%H_2_O_2_ solution. The tissue slide was incubated overnight at 4 °C with anti-ITGA2 antibody. Developed signals was detected using ABC Elite Kit (Vector, Burlingame, CA, USA) following the standard protocol. The tissue slides were counterstained and images were captured and examined under light microscope (Eclipse 80i Nikon Ins., Melville, NY, USA). The histoscore of each core in triplicate was calculated as we described [59].

### 4.12. Statistical Analysis

Comparisons among experimental and controls groups were performed using Welch-corrected unpaired *t*-test analysis (IBM SPSS Statistics for Windows, ver. 24 (IBM Coro., Armonk, NY, USA). Graphs were generated by Prism (GraphPad Software, Inc., La Jolla, CA, USA). Data were considered significant at *p*-value less than 0.05.

## 5. Conclusions

We report that exosomes-mediated transfer of ITGA2 alters, in part, AR-positive PCa cells to acquire more aggressive phenotypes by promoting their cell proliferation, migration, and invasion. Ectopic expression of *ITGA2* recapitulates the effect of exosome-associated ITGA2, induces EMT and increases the activity of FAK and ERK1/2 in transfected cells. Blocking the exosomal uptake by the MβCD inhibitor or knockdown of *ITGA2* rescued the effect of exosomes-mediated transfer of the integrin. This study highlights the importance of sorting out cargo contents during biogenesis of exosomes and the selectivity of target cells during their cellular uptake. In addition, it provides us with more knowledge of how AR-dependent PCa cells acquire more aggressive phenotypes through exosome-mediated transfer of cargo proteins derived from CRPC cells.

## Supplementary Materials

The following Supplementary Materials are available online at https://www.mdpi.com/2072-6694/12/8/2300/s1. Figure S1: Exosome-mediated transfer of ITGA2 promotes cell growth. Rc77T/E, Cr-2B, E006AA, and E006AA-hT cells were incubated with 10 µg/mL DU145-derived exosomes for three days. Migration, WST-8 and adhesion assays were performed in 6 replicates. * depicts significance at *p* < 0.05, Figure S2: Ectopic expression of ITGA2 in E006AA and C4-2B cells promotes cell migration. PCa cells transfected with ITGA2 and pcDNA3.1 as the control plasmid and a wound-healing assay was performed on two PCa cells for 24 and 48 h (A: C4-2B cells) and 12 and 24 h (B: E006AA cells) time periods. Each treatment was conducted in triplicate and images were acquired under inverted bright microscope, Figure S3: Exosomes-mediated transfer of ITGA2 changes PCa behaviors. C4-2B, CWR-R1ca and E006AA cells were incubated with exo^DU145^ and migration and invasion assays were performed (A). C4-2B and E006AA cells were transfected with ITGA2 and pcDNA3.1 plasmid as a vehicle and evaluated for ITGA2 expression by qPCR analysis (B) and clonogenic assay (C). Experiments conducted in triplicate and repeated at least thrice. * depicts significance at *p* < 0.05, Figure S4: Characterization and expression of ITGA2 in exosomes derived from plasma procured from PCa patients and cells, (A). Exosomes were characterized by immunoblotting (IB) analysis. IB analysis shows expression of calnexin as a cellular marker and CD9 and CD63 as exosomal markers in exosomes derived from PC-3, DU-145, C4-2B, and RWPE-1 cells and plasma collected from PCa patients (T1 and T2) or healthy individuals (N1 and N2), (B): Protein cell lysates were collected and the expression of cellular and exosomal contents of ITGA2 was evaluated in PCa cells using Western blot analysis, Figure S5: Exosomal-mediated transfer of ITGA2 in PCa cells, (A). Optimization of exosome-mediated transfer in PCa cells. About 20 μg protein lysates were collected from CWR-1Rca cells incubated with 0, 5 and 20 μg/mL Exo^PC-3^ for 48 h. The membranes were incubated with anti-ITGA2, anti-vimentin, anti-pERK1/2 and anti-GAPDH as a loading control protein, (B). Immunoblot (IB) analysis for C4-2B cells incubated with 20 μg/mL exo^PC-3^ for 24 h, (C). IB analysis of protein lysate collected from CWR-1Rca cells treated with or without 2.5 mM MβCD in the presence of 20 µg/mL exo^PC-3^. In addition, 20 μg protein lysate collected from CWR-1Rca cells incubated with 20 µg/mL Exo ^PC-3^ at different time points; 18, 24, and 48 h, (D). PC-3 cells were transduced with lentiviral particles carrying shRNA specific to ITGA2. C4-2B and LNCaP cells were incubated with exosomes isolated from the transduced PC-3 cells for 48 h, Figure S6: Ectopic expression of ITGA2 promotes aggressive phenotypes in PCa cells. C4-2B cells in addition to control E006AA cells were transfected by 2.5 µg ITGA2 or PCDNA3.1 as a control plasmid for 96 h. The expression of ITGA2, E-cadherin, vimentin, FAK and CMyc and the activity of ERK1/2 were evaluated in these cells, Figure S7: Expression of ITGA2 in exosomes isolated from the plasma of PCa patients. Exosomes were isolated from the sera of 14 PCa patients in addition to healthy subjects. Exosomal protein lysates were prepared and immunoblotting analysis was performed using anti-ITGA2, anti-CD81 and anti-Calnexin antibodies, Table S1: Clinical information of PCa patients from which sera were collected for evaluating the enrichment of ITGA2 in exosomes, Table S2: Characteristics of PCa cells used in the study.

## References

[1] Siegel R.L., Miller K.D., Jemal A. (2020). Cancer statistics, 2020. CA Cancer J. Clin..

[2] Endzeliņš E., Melne V., Kalniņa Z., Lietuvietis V., Riekstiņa U., Llorente A., Linē A. (2016). Diagnostic, prognostic and predictive value of cell-free miRNAs in prostate cancer: A systematic review. Mol. Cancer.

[3] Hutchinson L. (2014). Closing the Controversies Gap in Prostate Cancer. Nat. Rev. Clin. Oncol..

[4] Cheville J.C., Tindall D., Boelter C., Jenkins R., Lohse C.M., Pankratz V.S., Sebo T.J., Davis B., Blute M.L. (2002). Metastatic prostate carcinoma to bone: Clinical and pathologic features associated with cancer-specific survival. Cancer.

[5] Montgomery R.B., Mostaghel E.A., Vessella R., Hess D.L., Kalhorn T.F., Higano C.S., True L.D., Nelson P.S. (2008). Maintenance of intratumoral androgens in metastatic prostate cancer: A mechanism for castration-resistant tumor growth. Cancer Res..

[6] Chen C.D., Welsbie D.S., Tran C., Baek S.H., Chen R., Vessella R., Rosenfeld M.G., Sawyers C.L. (2004). Molecular determinants of resistance to antiandrogen therapy. Nat. Med..

[7] Debes J.D., Tindall D.J. (2004). Mechanisms of androgen-refractory prostate cancer. N. Engl. J. Med..

[8] Hu R., Dunn T.A., Wei S., Isharwal S., Veltri R.W., Humphreys E., Han M., Partin A.W., Vessella R.L., Isaacs W.B. (2009). Ligand-independent androgen receptor variants derived from splicing of cryptic exons signify hormone-refractory prostate cancer. Cancer Res..

[9] Stanbrough M., Bubley G.J., Ross K., Golub T.R., Rubin M.A., Penning T.M., Febbo P.G., Balk S.P. (2006). Increased expression of genes converting adrenal androgens to testosterone in androgen-independent prostate cancer. Cancer Res..

[10] Holzbeierlein J., Lal P., LaTulippe E., Smith A., Satagopan J., Zhang L., Ryan C., Smith S., Scher H., Scardino P. (2004). Gene expression analysis of human prostate carcinoma during hormonal therapy identifies androgen-responsive genes and mechanisms of therapy resistance. Am. J. Pathol..

[11] Agarwal N., Hutson T.E., Vogelzang N.J., Sonpavde G. (2010). Abiraterone acetate: A promising drug for the treatment of castration-resistant prostate cancer. Future Oncol..

[12] Feldman B.J., Feldman D. (2001). The development of androgen-independent prostate cancer. Nat. Rev. Cancer.

[13] Kelly S.P., Anderson W.F., Rosenberg P.S., Cook M.B. (2018). Past, current, and future incidence rates and burden of metastatic prostate cancer in the united States. Eur. Urol. Focus.

[14] Antonarakis E.S. (2016). Current understanding of resistance to abiraterone and enzalutamide in advanced prostate cancer. Clin. Adv. Hematol. Oncol..

[15] Bonkhoff H. (2018). Estrogen receptor signaling in prostate cancer: Implications for carcinogenesis and tumor progression. Prostate.

[16] Abd Elmageed Z.Y., Moroz K., Srivastav S.K., Fang Z., Crawford B.E., Moparty K., Thomas R., Abdel-Mageed A.B. (2013). High circulating estrogens and selective expression of ERbeta in prostate tumors of Americans: Implications for racial disparity of prostate cancer. Carcinogenesis.

[17] Di Zazzo E., Galasso G., Giovannelli P., di Donato M., Bilancio A., Perillo B., Sinisi A.A., Migliaccio A., Castoria G. (2019). Estrogen receptors in epithelial-mesenchymal transition of prostate cancer. Cancers.

[18] Cox T.R., Rumney R.M.H., Schoof E.M., Perryman L., Hoye A.M., Agrawal A., Bird D., Latif N.A., Forrest H., Evans H.R. (2015). The hypoxic cancer secretome induces pre-metastatic bone lesions through lysyl oxidase. Nature.

[19] Steinbichler T.B., Dudas J., Riechelmann H., Skvortsova I.I. (2017). The role of exosomes in cancer metastasis. Semin. Cancer Biol..

[20] Hoshino A., Costa-Silva B., Shen T.L., Rodrigues G., Hashimoto A., Mark M.T., Molina H., Kohsaka S., di Giannatale A., Ceder S. (2015). Tumour exosome integrins determine organotropic metastasis. Nature.

[21] Kim H., Lee S., Shin E., Seong K.M., Jin Y.W., Youn H., Youn B. (2020). The emerging roles of exosomes as EMT regulators in cancer. Cells.

[22] Xiao L., Wang Y., Xu K., Hu H., Xu Z., Wu D., Wang Z., You W., Ng C.F., Yu S. (2018). Nuclear receptor LRH-1 functions to promote castration-resistant growth of prostate cancer via its promotion of intratumoral androgen biosynthesis. Cancer Res..

[23] Seguin L., Desgrosellier J.S., Weis S.M., Cheresh D.A. (2015). Integrins and cancer: Regulators of cancer stemness, metastasis, and drug resistance. Trends Cell Biol..

[24] Goel H.L., Li J., Kogan S., Languino L.R. (2008). Integrins in prostate cancer progression. Endocr. Relat. Cancer.

[25] Elices M.J., Hemler M.E. (1989). The human integrin VLA-2 is a collagen receptor on some cells and a collagen/laminin receptor on others. Proc. Natl. Acad. Sci. USA.

[26] Ren D., Zhao J., Sun Y., Li D., Meng Z., Wang B., Fan P., Liu Z., Jin X., Wu H. (2019). Overexpressed ITGA2 promotes malignant tumor aggression by up-regulating PD-L1 expression through the activation of the STAT3 signaling pathway. J. Exp. Clin. Cancer Res..

[27] Wang Q., Cao T., Guo K., Zhou Y., Liu H., Pan Y., Hou Q., Nie Y., Fan D., Lu Y. (2020). Regulation of integrin subunit Alpha 2 by miR-135b-5p modulates chemoresistance in gastric cancer. Front. Oncol..

[28] Skrzypek K., Kot M., Konieczny P., Nieszporek A., Kusienicka A., Lasota M., Bobela W., Jankowska U., Kedracka-Krok S., Majka M. (2020). SNAIL promotes metastatic behavior of rhabdomyosarcoma by increasing EZRIN and AKT expression and regulating MicroRNA networks. Cancers.

[29] Elmageed Z.Y.A., Yang Y., Thomas R., Ranjan M., Mondal D., Moroz K., Fang Z., Rezk B.M., Moparty K., Sikka S.C. (2014). Neoplastic reprogramming of patient-derived adipose stem cells by prostate cancer cell-associated exosomes. Stem Cells.

[30] Shephard A.P., Yeung V., Clayton A., Webber J.P. (2017). Prostate cancer exosomes as modulators of the tumor microenvironment. J. Cancer Metastasis Treat..

[31] Saber S.H., Ali H.E.A., Gaballa R., Gaballah M., Ali H.I., Zerfaoui M., Abd Elmageed Z.Y. (2020). Exosomes are the driving force in preparing the soil for the metastatic seeds: Lessons from the prostate cancer. Cells.

[32] Hooker S.E., Woods-Burnham L., Bathina M., Lloyd S., Gorjala P., Mitra R., Nonn L., Kimbro K.S., Kittles R.A. (2019). Genetic ancestry analysis reveals misclassification of commonly used cancer cell lines. Cancer Epidemiol. Biomark. Prev..

[33] Karpf A.R., Bai S., James S.R., Mohler J.L., Wilson E.M. (2009). Increased expression of androgen receptor coregulator MAGE-11 in prostate cancer by DNA hypomethylation and cyclic AMP. Mol. Cancer Res..

[34] Obinata D., Takayama K., Urano T., Murata T., Ikeda K., Horie-Inoue K., Ouchi Y., Takahashi S., Inoue S. (2012). ARFGAP3, an androgen target gene, promotes prostate cancer cell proliferation and migration. Int. J. Cancer.

[35] Hamidi H., Ivaska J. (2018). Every step of the way: Integrins in cancer progression and metastasis. Nat. Rev. Cancer.

[36] Lu H., Wang T., Li J., Fedele C., Liu Q., Zhang J., Jiang Z., Jain D., Iozzo R.V., Violette S.M. (2016). Alphavbeta6 integrin promotes castrate-resistant prostate cancer through JNK1-mediated activation of androgen receptor. Cancer Res..

[37] Chuang Y.C., Wu H.Y., Lin Y.L., Tzou S.C., Chuang C.H., Jian T.Y., Chen P.R., Chang Y.C., Lin C.H., Huang T.H. (2018). Blockade of ITGA2 induces apoptosis and inhibits cell migration in gastric cancer. Biol. Proced. Online.

[38] Ziaee S., Chung L.W. (2014). Induction of integrin alpha2 in a highly bone metastatic human prostate cancer cell line: Roles of RANKL and AR under three-dimensional suspension culture. Mol. Cancer.

[39] Hall C.L., Dubyk C.W., Riesenberger T.A., Shein D., Keller E.T., van Golen K.L. (2008). Type I collagen receptor (alpha2beta1) signaling promotes prostate cancer invasion through RhoC GTPase. Neoplasia.

[40] Krishn S.R., Singh A., Bowler N., Duffy A.N., Friedman A., Fedele C., Kurtoglu S., Tripathi S.K., Wang K., Hawkins A. (2019). Prostate cancer sheds the alphavbeta3 integrin in vivo through exosomes. Matrix Biol..

[41] Lu H., Bowler N., Harshyne L.A., Hooper D.C., Krishn S.R., Kurtoglu S., Fedele C., Liu Q., Tang H.Y., Kossenkov A.V. (2018). Exosomal alphavbeta6 integrin is required for monocyte M2 polarization in prostate cancer. Matrix Biol..

[42] Singh A., Fedele C., Lu H., Nevalainen M.T., Keen J.H., Languino L.R. (2016). Exosome-mediated transfer of alphavbeta3 integrin from tumorigenic to nontumorigenic cells promotes a migratory phenotype. Mol. Cancer Res..

[43] Bijnsdorp I.V., Geldof A.A., Lavaei M., Piersma S.R., van Moorselaar R.J., Jimenez C.R. (2013). Exosomal ITGA3 interferes with non-cancerous prostate cell functions and is increased in urine exosomes of metastatic prostate cancer patients. J. Extracell. Vesicles.

[44] Koumangoye R.B., Sakwe A.M., Goodwin J.S., Patel T., Ochieng J. (2011). Detachment of breast tumor cells induces rapid secretion of exosomes which subsequently mediate cellular adhesion and spreading. PLoS ONE.

[45] Svensson K.J., Christianson H.C., Wittrup A., Bourseau-Guilmain E., Lindqvist E., Svensson L.M., Morgelin M., Belting M. (2013). Exosome uptake depends on ERK1/2-heat shock protein 27 signaling and lipid Raft-mediated endocytosis negatively regulated by caveolin-1. J. Biol. Chem..

[46] McAtee C.O., Booth C., Elowsky C., Zhao L., Payne J., Fangman T., Caplan S., Henry M.D., Simpson M.A. (2019). Prostate tumor cell exosomes containing hyaluronidase Hyal1 stimulate prostate stromal cell motility by engagement of FAK-mediated integrin signaling. Matrix Biol..

[47] Dai J., Escara-Wilke J., Keller J.M., Jung Y., Taichman R.S., Pienta K.J., Keller E.T. (2019). Primary prostate cancer educates bone stroma through exosomal pyruvate kinase M2 to promote bone metastasis. J. Exp. Med..

[48] Li X.L., Liu L., Li D.D., He Y.P., Guo L.H., Sun L.P., Liu L.N., Xu H.X., Zhang X.P. (2017). Integrin beta4 promotes cell invasion and epithelial-mesenchymal transition through the modulation of Slug expression in hepatocellular carcinoma. Sci. Rep..

[49] Ryu J., Koh Y., Park H., Kim D.Y., Kim D.C., Byun J.M., Lee H.J., Yoon S.S. (2016). Highly expressed integrin-alpha8 induces epithelial to mesenchymal transition-like features in multiple myeloma with early relapse. Mol. Cells.

[50] Pontes-Junior J., Reis S.T., Dall’Oglio M., de Oliveira L.C.N., Cury J., Carvalho P.A., Ribeiro-Filho L.A., Leite K.R.M., Srougi M. (2009). Evaluation of the expression of integrins and cell adhesion molecules through tissue microarray in lymph node metastases of prostate cancer. J. Carcinog..

[51] Bonkhoff H., Stein U., Remberger K. (1993). Differential expression of alpha 6 and alpha 2 very late antigen integrins in the normal, hyperplastic, and neoplastic prostate: Simultaneous demonstration of cell surface receptors and their extracellular ligands. Hum. Pathol..

[52] Ding W., Fan X.L., Xu X., Huang J.Z., Xu S.H., Geng Q., Li R., Chen D., Yan G.R. (2015). Epigenetic silencing of ITGA2 by MiR-373 promotes cell migration in breast cancer. PLoS ONE.

[53] Dong J., Wang R., Ren G., Li X., Wang J., Sun Y., Liang J., Nie Y., Wu K., Feng B. (2017). HMGA2-FOXL2 axis regulates metastases and epithelial-to-mesenchymal transition of chemoresistant gastric cancer. Clin. Cancer Res..

[54] Ivell R., Teerds K., Hoffman G.E. (2014). Proper application of antibodies for immunohistochemical detection: Antibody crimes and how to prevent them. Endocrinology.

[55] Allott E.H., Geradts J., Sun X., Cohen S.M., Zirpoli G.R., Khoury T., Bshara W., Chen M., Sherman M.E., Palmer J.R. (2016). Intratumoral heterogeneity as a source of discordance in breast cancer biomarker classification. Breast Cancer Res..

[56] Robinson S.D., Hodivala-Dilke K.M. (2011). The role of beta3-integrins in tumor angiogenesis: Context is everything. Curr. Opin. Cell Biol..

[57] Ali H.E.A., Lung P.Y., Sholl A.B., Gad S.A., Bustamante J.J., Ali H.I., Rhim J.S., Deep G., Zhang J., Abd Elmageed Z.Y. (2018). Dysregulated gene expression predicts tumor aggressiveness in African-American prostate cancer patients. Sci. Rep..

[58] Mirzapoiazova T., Lennon F.E., Mambetsariev B., Allen M., Riehm J., Poroyko V.A., Singleton P.A. (2015). Extracellular vesicles from caveolin-enriched microdomains regulate hyaluronan-mediated sustained vascular integrity. Int. J. Cell Biol..

[59] Elmageed Z.Y.A., Moore R.F., Tsumagari K., Lee M.M., Sholl A.B., Friedlander P., Al-Qurayshi Z., Hassan M., Wang A.R., Boulares H.A. (2018). Prognostic role of BRAF(V600E) cellular localization in melanoma. J. Am. Coll. Surg..

