# Supplementary material for: Exosomes-Mediated Transfer of Itga2 Promotes Migration and Invasion of Prostate Cancer Cells by Inducing Epithelial-Mesenchymal Transition
Source: Cancers (Basel). 2020 Aug 15;12(8):2300. doi: 10.3390/cancers12082300 (PMC7466113; doi:10.3390/cancers12082300)
Supplement: Supplementary file 1 [file cancers-12-02300-s001.pdf]

cancers

Article

Exosomes-Mediated Transfer of Itga2 Promotes Migration and Invasion of Prostate Cancer Cells by Inducing Epithelial-Mesenchymal Transition

Rofaida Gaballa, Hamdy EA Ali, Mohamed O. Mahmoud, Johng S. Rhim, Hamed I. Ali, Heba F. Salem, Mohammad Saleem, Mohamed A. Kandeil, Stefan Ambs and Zakaria Y. Abd Elmageed

Supplementary Materials

1. Methods

1.1. Cell adhesion assay

Ninety-six well plate was coated with 40µg/mL type I collagen (Sigma-Aldrich, St. Louis, MO, USA) in PBS and kept for 12 h at 4 °C, air dried, and rinsed once with PBS. C4-2B and E006AA cells were incubated with 15 µg/mL exosomes-derived from DU145 cells for 48 h following serum deprivation for 8 h. Cells were detached by adding 10mmol/L EDTA in DMEM, washed, and about 2 × 10^4 cells/well were plated in quadruplicate into the collagen-coated 96-well plate in SFM containing 0.1% BSA. Cells in three wells of the quadruplicate were allowed to adhere on collagen I–coated surface for 20 min, followed by four intensive washes to remove non-adherent cells. The fourth one of the quadruplicate wells was used as a control for cell count. Adhered cells incubated then with 10 µL of WST-8 reagent for another 2h. The developed color was measured at 450 nm by accuSkan FC plate reader (Fisher Scientific, Hampton, NH, USA).

Table 1. Clinical information of PCa patients from which plasma were collected for evaluating the enrichment of ITGA2 in exosomes.

Case#	Age	Race	GS	Stage	Recurrence	Type of 1st recurrence
**1**	72	African American	6	IIB	Yes	Distant to bone
**2**	55	African American	6	I	No	No recurrence
**3**	55	African American	6	IIB	No	No recurrence
**4**	59	European American	6	I	No	No recurrence
**5**	66	European American	6	IIA	Yes	Local recurrence
**6**	69	European American	6	I	Yes	Regional LNS
**7**	56	European American	6	IIB	No	No recurrence
**8**	79	European American	9	IIB	Unknown	Unknown
**9**	71	African American	8	IIB	No	No recurrence
**10**	74	African American	8	IV	No	No recurrence
**11**	64	European American	9	IIB	No	No recurrence
**12**	61	African American	8	IIA	Yes	Local recurrence
**13**	63	African American	9	IV	No	No recurrence
**14**	63	European American	9	IIB	No	No recurrence

Table 2. Characteristics of PCa cells used in the study.

Cell line	Description	AR	ERα	ERβ	CRPC	Ref.
PC-3	Metastatic PCa cells derived from bone of 62-y old white patient	−	+	+	+	[1]
DU145	Metastatic PCa cells derived from brain of 69-y old white patient	−	−	+	-	[1]
CWR-R1ca	PCa cells derived from the castration recurrent CWR22 xenograft tumors and fibroblasts were depleted from these cells	+			+	[2]
LNCaP	PCa cells derived from lymph node of 52-y old patient	+	+	+	−	[1]
C4-2B	PCa cells derived from LNCaP C4-2 cells	+	−	+	−	[3]
MDA-PCa-2b	Metastatic PCa cells derived from bone of 63-y old black patient	+	−	+	−	[4]
RC77 T/E	PCa cells derived radical prostatectomy of 63-y old black patient at Gleason score 7	+			−	[5]
RC77 N/E	Transformed non-malignant cells derived from adjacent normal region of 63-y old black patient at Gleason score 7	+			−	[5]
RWPE-1	Transformed non-malignant prostate epithelial cells derived from 54-y white man.	−	+	+	−	[6]

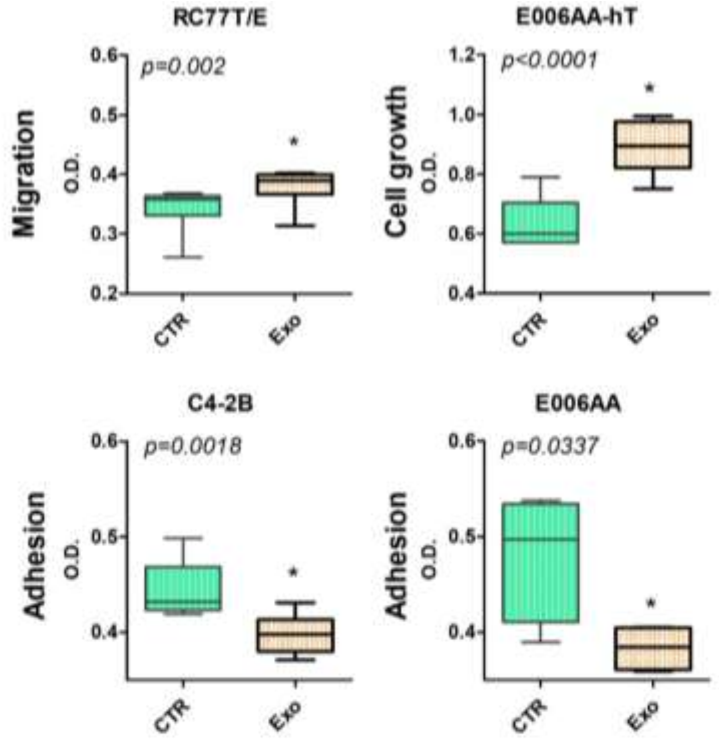

Figure 1. Exosomes-mediated transfer of ITGA2 promotes cell growth. Rc77T/E, C4-2B, E006AA and E006AA-hT cells were incubated with 10 μg/mL DU145-derived exosomes for 24-72h based on the assay. Migration, WST-8 and adhesion assays were performed in 6 replicates. * depicts significance at $p < 0.05$.

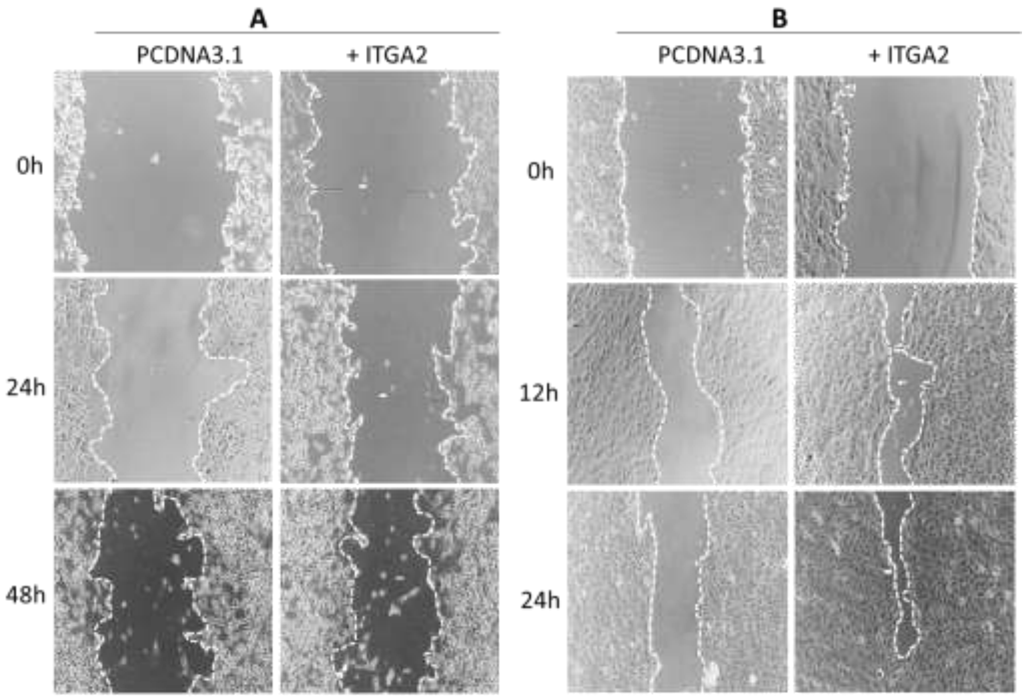

Figure 2. Ectopic expression of ITGA2 in E006AA and C4-2B cells promotes cell migration. PCa cells transfected with ITGA2 and pcDNA3.1 as control plasmid and wound-healing assay was performed on two PCa cells for 24 & 48h (**A**: C4-2B cells) and 12 & 24h (**B**: E006AA cells) time periods. Each treatment was conducted in triplicates and images were acquired under inverted bright microscope.

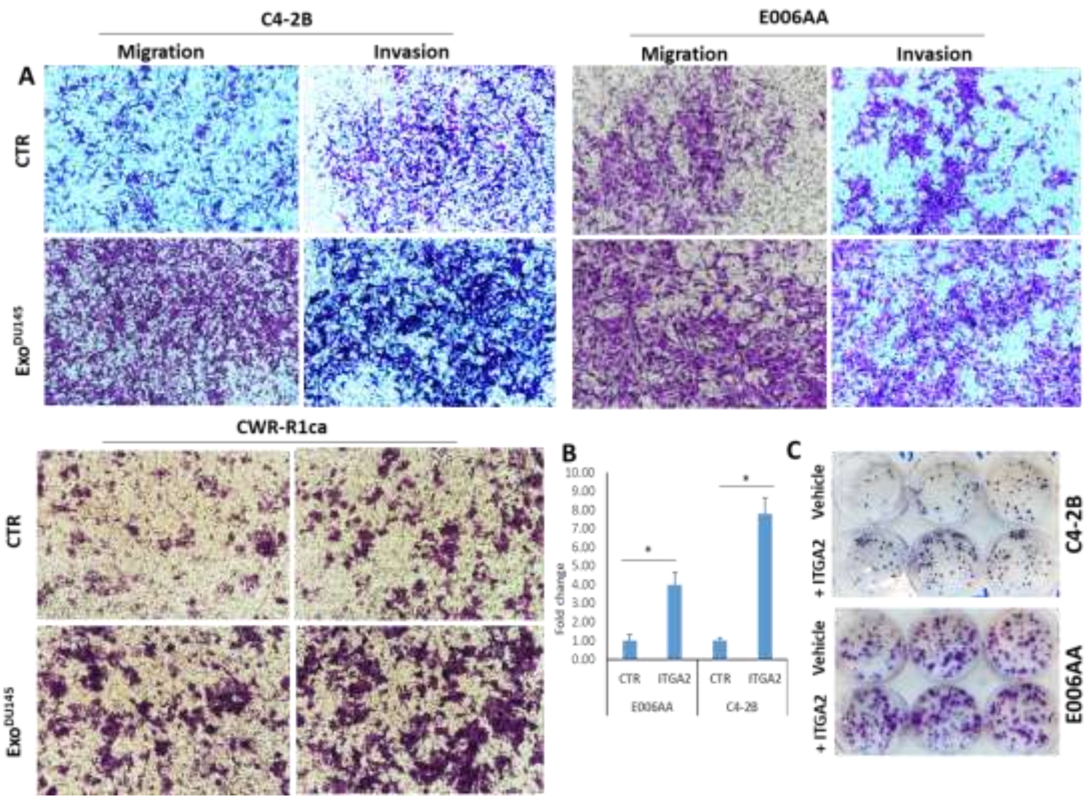

Figure 3. Exosomes-mediated transfer of ITGA2 changes PCa behaviors. C4-2B, CWR-R1ca and E006AA cells were incubated with exo^{DU145} and migration and invasion assays were performed (**A**). C4-2B and E006AA cells were transfected with ITGA2 and pcDNA3.1 plasmids as a vehicle and evaluated for ITGA2 expression by qPCR analysis (**B**) and clonogenic assay (**C**). Experiments conducted in triplicated and repeated at least thrice. * depicts significance at $p < 0.05$.

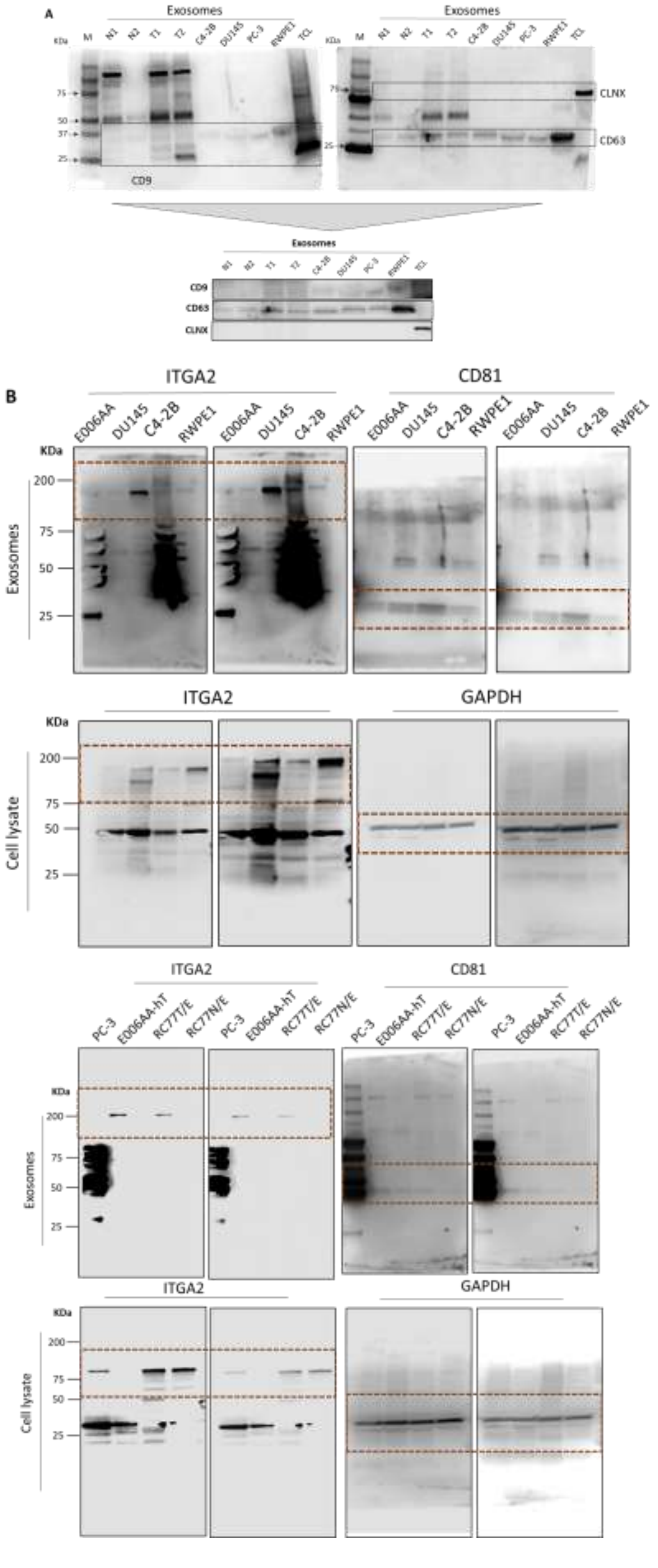
A
Exosomes
KDa
M
N1
N2
T1
T2
C4-2B
DU145
PC-3
RWPE1
TCL
75
50
37
25
CD9
CLNX
CD63
B
ITGA2
CD81
E006AA
DU145
C4-2B
RWPE1
200
75
50
25
Exosomes
GAPDH
Cell lysate
PC-3
E006AA-hT
RC77T/E
RC77N/E

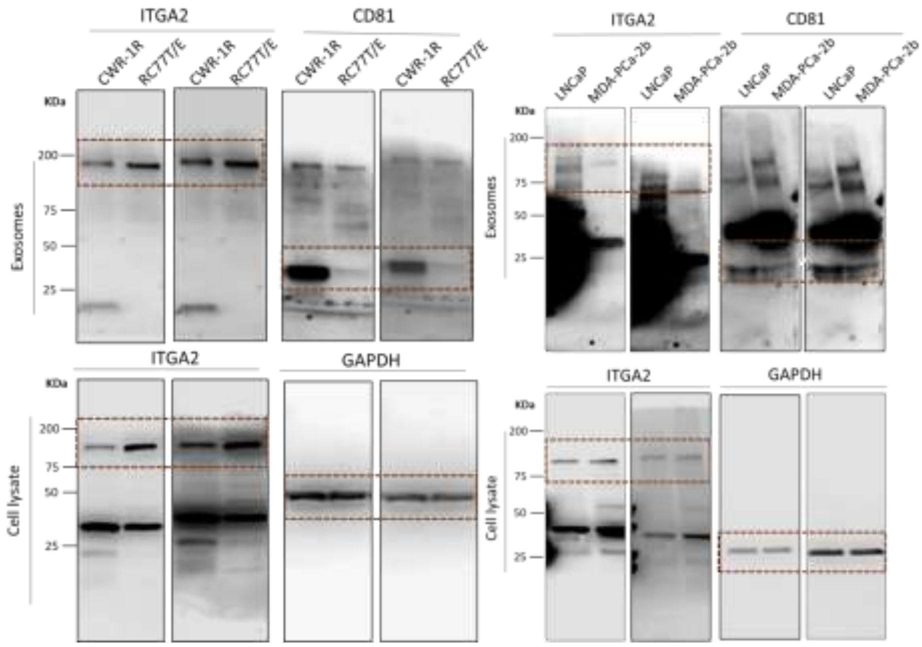

Figure S4. Characterization and expression of ITGA2 in exosomes derived from plasma procured from PCa patients and cells. (**A**) Exosomes were characterized by immunoblotting (IB) analysis. IB analysis shows expression of calnexin as a cellular marker and CD9 and CD63 as exosomal markers in exosomes derived from PC-3, DU-145, C4-2B and RWPE-1 cells and plasma collected from PCa patients (T1 & T2) or healthy individuals (N1 & N2). (**B**) Protein cell lysates were collected and the expression of cellular and exosomal contents of ITGA2 was evaluated in PCa cells using Western blot analysis.

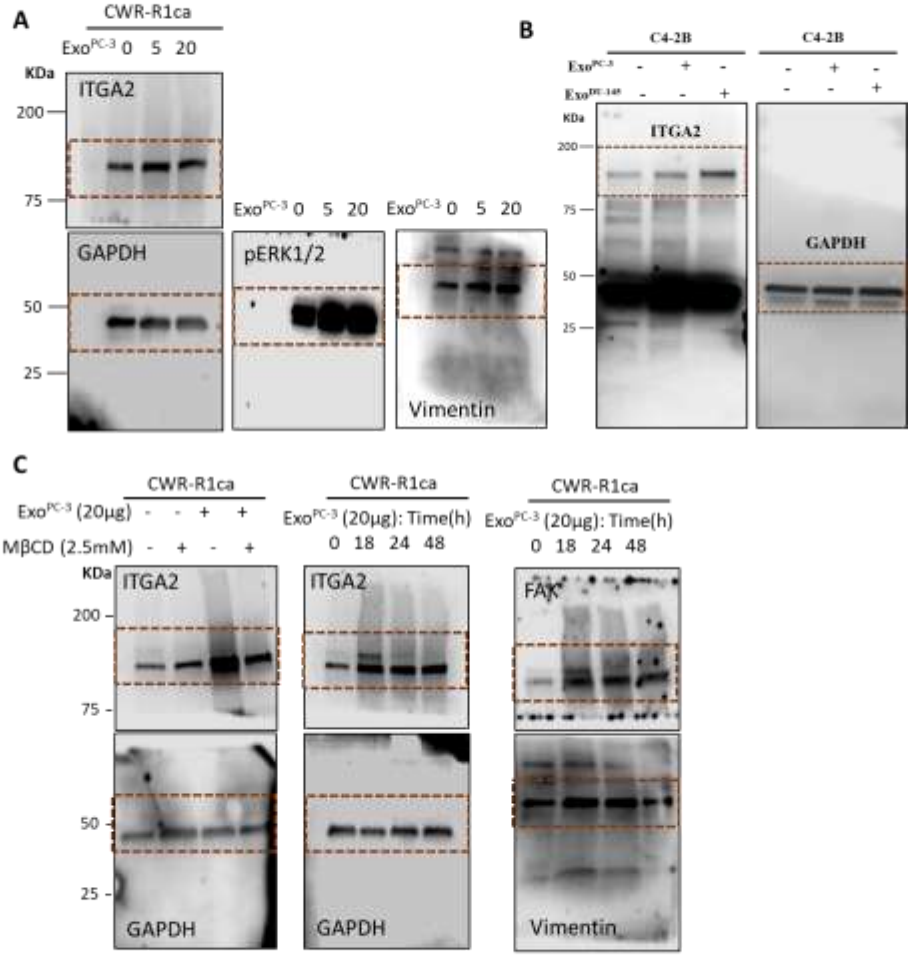

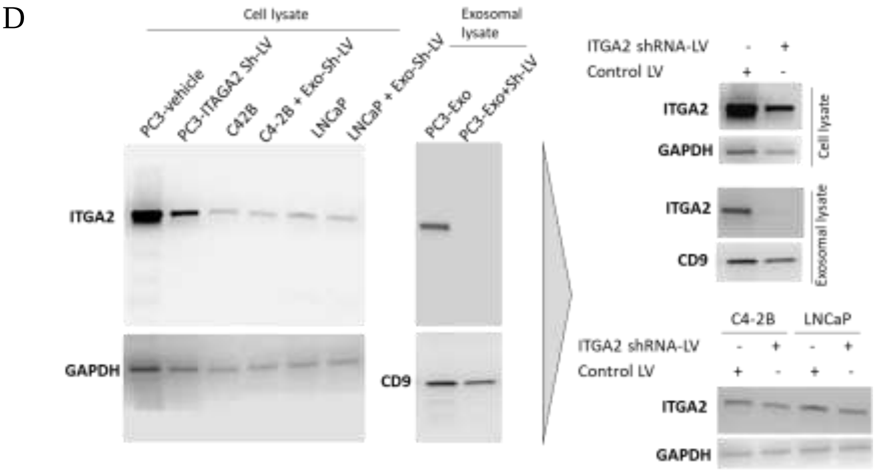

Figure S5. Exosomal-mediated transfer of ITGA2 in PCa cells**.** (**A**). Optimization of exosomes-mediated transfer in PCa cells. About 20µg protein lysates were collected from CWR-1Rca cells incubated with 0, 5 and 20 µg/mL Exo^{PC-3} for 48h. The membranes were incubated with anti-ITGA2, anti-vimentin, anti-pERK1/2 and anti-GAPDH as a loading control protein. (**B**). Immunoblot (IB) analysis for C4-2B cells incubated with 20µg/mL exo^{PC-3} for 24 h. **C**. IB analysis of protein lysate collected from CWR-1Rca cells treated with or without 2.5 mM MβCD in the presence of 20 µg/mL exo^{PC-3}. In addition, 20µg protein lysate collected from CWR-1Rca cells incubated with 20 µg/mL Exo^{PC-3} at different time points; 18, 24 and 48 h. **D**: PC-3 cells were transduced with lentiviral particles carrying shRNA specific to ITGA2. C4-2B and LNCaP cells were incubated with exosomes isolated from the transduced PC-3 cells for 24 h.

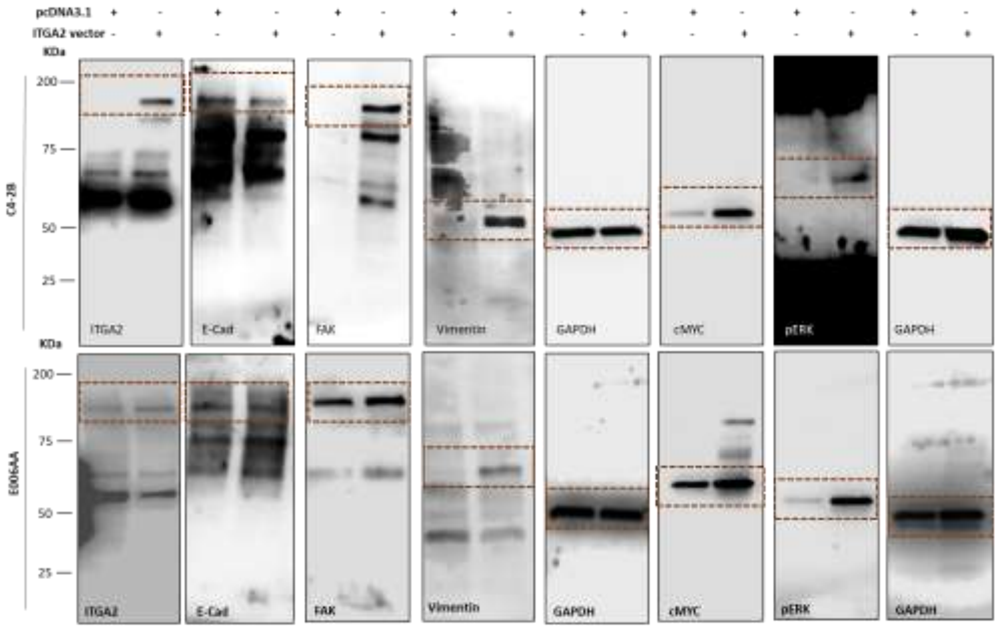

Figure S6. Ectopic expression of ITGA2 promotes aggressive phenotypes in PCa cells. C4-2B cells in addition to control E006AA cells were transfected by 2.5µg ITGA2 or PCDNA3.1 as a control plasmid for 96 h. The expression of ITGA2, E-cadherin, vimentin, FAK and CMyc and the activity of ERK1/2 were evaluated in these cells.

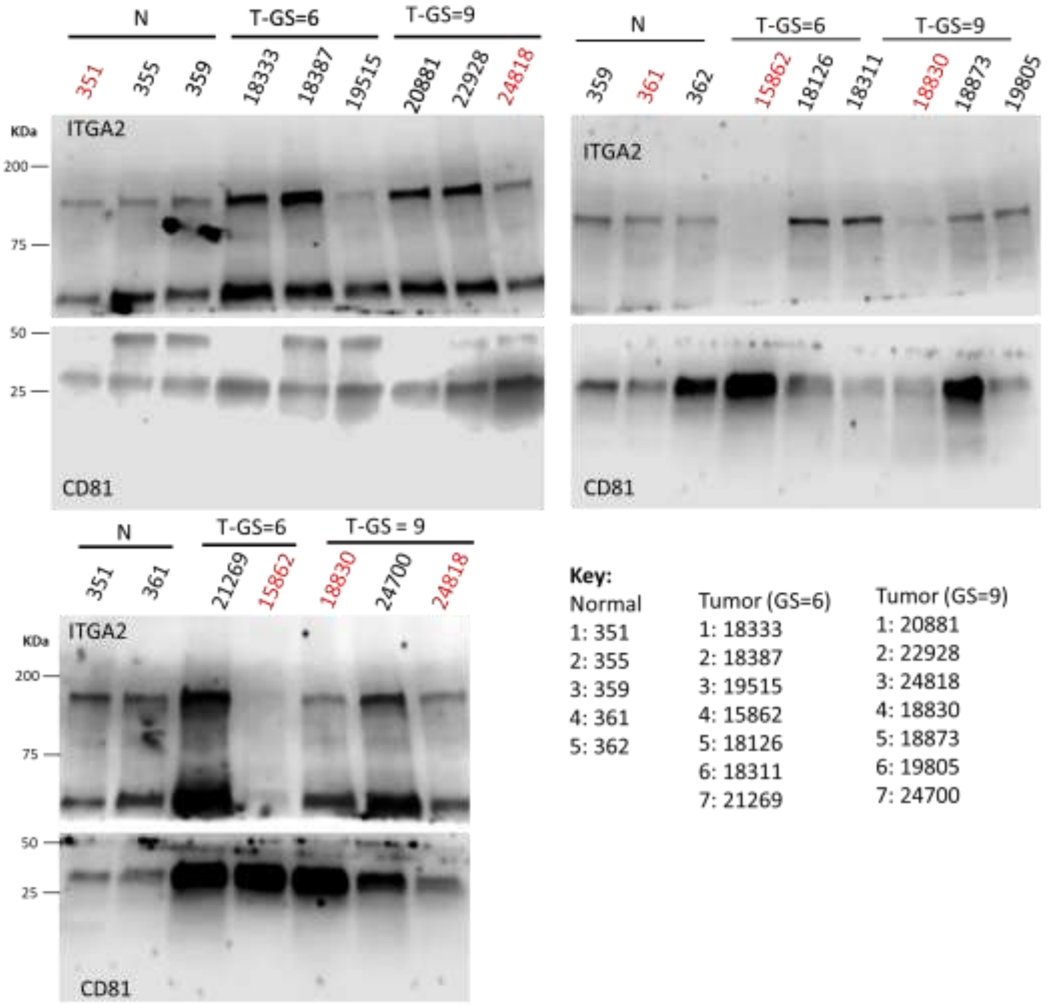

Figure S7. Expression of ITGA2 in exosomes isolated from the plasma of PCa patients. Exosomes were isolated from the sera of 14 PCa patients in addition to healthy subjects. Exosomal protein lysates were prepared and immunoblotting analysis was performed using anti-ITGA2, anti-CD81 and anti-Calnexin antibodies.

References

1. Lau, K.M.; LaSpina, M.; Long, J.; Ho, S.M. Expression of estrogen receptor (ER)-alpha and ER-beta in normal and malignant prostatic epithelial cells: regulation by methylation and involvement in growth regulation. *Cancer Res*. **2000**, 60, 3175–3182.
2. Li, Y.; Chan, S.C.; Brand, L.J.; Hwang, T.H.; Silverstein, K.A.; Dehm, S.M. Androgen receptor splice variants mediate enzalutamide resistance in castration-resistant prostate cancer cell lines. *Cancer Res*. 2013, 73, 483–489.
3. Cheung, C.P.; Yu, S.; Wong, K.B.; Chan, L.W.; Lai, F.M.; Wang, X.; Suetsugi, M.; Chen, S.; Chan, F.L. Expression and functional study of estrogen receptor-related receptors in human prostatic cells and tissues, *J. Clin. Endocrinol. Metab*. **2005**, *90*, 1830–1844.
4. Mishra, S.; Tai, Q.; Gu, X.; Schmitz, J.; Poullard, A.; Fajardo, R.J.; Mahalingam, D.; Chen, X.; Zhu, X.; Sun, L.Z. Estrogen and estrogen receptor alpha promotes malignancy and osteoblastic tumorigenesis in prostate cancer. *Oncotarget* **2015**, *6*, 44388–44402.
5. Theodore, S.; Sharp, S.; Zhou, J.; Turner, T.; Li, H.; Miki, J.; Ji, Y.; Patel, V.; Yates, C.; Rhim, J.S. Establishment and characterization of a pair of non-malignant and malignant tumor derived cell lines from an African American prostate cancer patient. *Int. J. Oncol.* **2010**, *37*, 1477–1482.
6. Raza, S.; Meyer, M.; Goodyear, C.; Hammer, K.D.P.; Guo, B.; Ghribi, O. The cholesterol metabolite 27-hydroxycholesterol stimulates cell proliferation via ERbeta in prostate cancer cells. *Cancer Cell Int.* **2017**, *17*, 52.
